# Metal-dependent allosteric activation and inhibition on the same molecular scaffold: the copper sensor CopY from *Streptococcus pneumoniae*†
†Electronic supplementary information (ESI) available. See DOI: 10.1039/c7sc04396a


**DOI:** 10.1039/c7sc04396a

**Published:** 2017-11-09

**Authors:** Hendrik Glauninger, Yifan Zhang, Khadine A. Higgins, Alexander D. Jacobs, Julia E. Martin, Yue Fu, H. Jerome Coyne, 3rd, Kevin E. Bruce, Michael J. Maroney, David E. Clemmer, Daiana A. Capdevila, David P. Giedroc

**Affiliations:** a Department of Chemistry , Indiana University , Bloomington , IN 47405-7102 , USA . Email: giedroc@indiana.edu ; Email: dacapdev@iu.edu ; Tel: +1-812-856-3178 ; Tel: +1-812-856-6398; b Department of Molecular and Cellular Biochemistry , Indiana University , Bloomington , IN 47405 , USA; c Department of Chemistry , Salve Regina University , Newport , RI 02840 , USA; d Department of Biology , Indiana University , Bloomington , IN 47405 , USA; e Department of Chemistry , University of Massachusetts , Amherst , MA 01003 , USA

## Abstract

Resistance to copper (Cu) toxicity in the respiratory pathogen *Streptococcus pneumoniae* is regulated by the Cu-specific metallosensor CopY. CopY is structurally related to the antibiotic-resistance regulatory proteins MecI and BlaI from *Staphylococcus aureus*, but is otherwise poorly characterized. Here we employ a multi-pronged experimental strategy to define the *Spn* CopY coordination chemistry and the unique mechanism of allosteric activation by Zn(ii) and allosteric inhibition by Cu(i) of *cop* promoter DNA binding. We show that Zn(ii) is coordinated by a subunit-bridging 3S 1H_2_O complex formed by the same residues that coordinate Cu(i), as determined by X-ray absorption spectroscopy and ratiometric pulsed alkylation-mass spectrometry (rPA-MS). Apo- and Zn-bound CopY are homodimers by small angle X-ray scattering (SAXS); however, Zn stabilizes the dimer, narrows the conformational ensemble of the apo-state as revealed by ion mobility-mass spectroscopy (IM-MS), and activates DNA binding *in vitro* and in cells. In contrast, Cu(i) employs the same Cys pair to form a subunit-bridging, kinetically stable, multi-metallic Cu·S cluster (*K*_Cu_ ≈ 10^16^ M^–1^) that induces oligomerization beyond the dimer as revealed by SAXS, rPA-MS and NMR spectroscopy, leading to inhibition of DNA binding. These studies suggest that CopY employs conformational selection to drive Zn-activation of DNA binding, and a novel Cu(i)-mediated assembly mechanism that dissociates CopY from the DNA *via* ligand exchange-catalyzed metal substitution, leading to expression of Cu resistance genes. Mechanistic parallels to antibiotic resistance repressors MecI and BlaI are discussed.

## Abstract 2

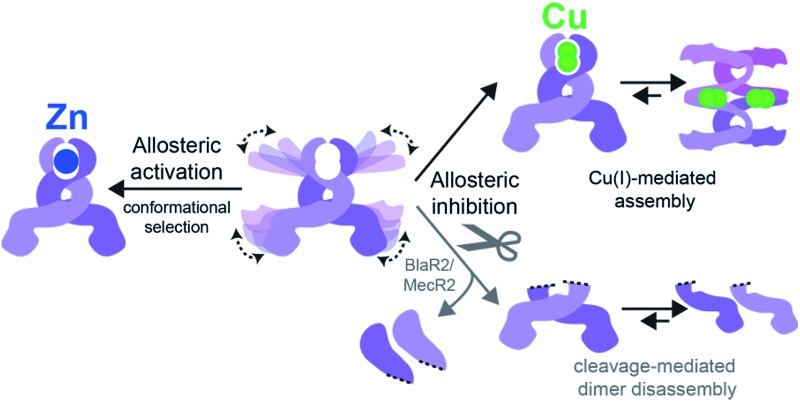
The dynamics and marginal stability of CopY enable allosteric activation of DNA binding by Zn(ii) and inhibition by Cu(i).

## Introduction

Transition metals are harnessed by enzymes as critical components of both their catalytic function and structural stability. It is estimated that approximately a quarter of all proteins require metals as essential cofactors.^1^ The same reactivity responsible for their enzymatic utility also serves to make transition metals potentially toxic, and as a result, all cells encode machinery that regulates the bioavailability of transition metals in cells *via* both uptake and efflux mechanisms.^2^ Metalloregulation of the transcription of genes encoding metal uptake and export transporters is particularly important for colonization and adaptation of pathogens in the vertebrate host. This adaptation features microbial defense mechanisms that either limit the availability of required transition metals in a process termed nutritional immunity,^3^ or induce metal intoxication,^4^ depending on the microenvironmental niche.^5^ In the case of Cu, there is emerging evidence to suggest that Cu is actively used to kill pathogenic invaders.^6^–^9^ Prokaryotic metalloregulatory proteins control the expression of genes responsible for the maintenance of appropriate intracellular metal levels *via* allosteric regulation of the DNA operator–promoter binding by metals, and thus are metal-regulated transcriptional “switches”.^10^–^12^

*Streptococcus pneumoniae* is a commensal Gram-positive respiratory pathogen that colonizes the upper respiratory tract in humans and is a primary cause globally of bacterial pneumonia,^13^ with ≈30% of severe *S. pneumoniae* infections possessing resistance to at least one clinically important antibiotic. In the serotype 2 *Streptococcus pneumoniae* D39 strain, CopY is a Cu(i)-sensing metalloregulatory repressor that regulates the expression of the *cop* operon, encoding CopY, the membrane-anchored copper chaperone CupA,^14^ and the Cu-effluxer CopA.^13^ The *cop* operon contributes to virulence of *S. pneumoniae* in a lung infection mouse model where the Δ*cupA* and Δ*copA* strains are attenuated for virulence; furthermore, the operon is induced in the nasopharynx and the lungs and as such, a Δ*copA* strain exhibits poor colonization of the nasopharynx.^13^ Transcriptional analysis by real time quantitative PCR (qRT-PCR)^13^ and a transcriptomic analysis of a Δ*copY* deletion strain^14^ collectively reveal that the *cop* operon is autoregulated by CopY. However, the details of regulatory mechanism used by *Spn* CopY in the pneumococcus remain unclear.

**Fig. 1:**
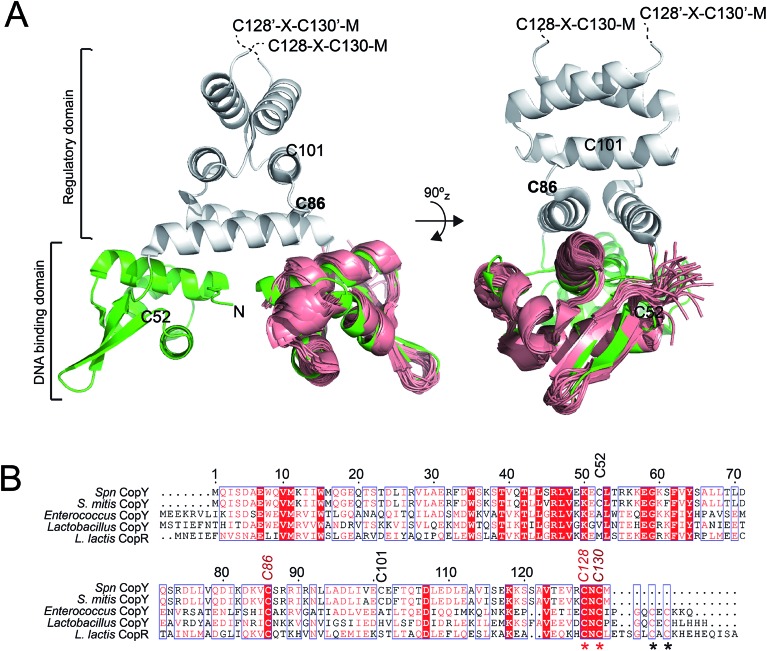
Dimeric CopY structural model. (A) Ribbon representation of the superposition of the solution structure bundle of the *L. lactis* CopR DNA binding domain (*salmon*)^17^ on the crystallographic structure of *S. aureus* BlaI (*green*, DNA-binding domain; *grey*, regulatory domain).^24^ The approximate positions of the five *Spn* CopY Cys are indicated, with the C128–C130 pair just beyond the solved structure of the BlaI determined by a multiple sequence alignment (not shown). (B) Multiple sequence alignment of CopY proteins from different bacteria. The conserved Cys residue proposed to be involved in copper and zinc binding are highlighted with asterisks. Note that *S. pneumoniae* CopY only contains two of the four Cys residues near the C-terminus. The C-terminus of the *L. lactis* CopR DNA binding domain model (see panel A) is E74 (T69 in *Spn* CopY), with the last residue of the β2 strand R70 (S65 in *Spn* CopY). K119 in the *Spn* CopY sequence defines the C-terminus of the BlaI model^24^ shown in panel A.

CopY is representative of a small family of copper-specific metalloregulatory proteins, initially characterized in *Enterococcus hirae*.^15^,^16^ CopY is a Cu(i)-sensing repressor that binds to one or more *cop* box sequences in the promoter and transcriptionally regulates the expression of downstream genes in response to cellular Cu(i) toxicity. The DNA-binding domain is a canonical winged-helical motif, as determined by the solution structure of the N-terminal domain of *Lactococcus lactis* CopR.^17^ CopY is proposed to be member of methicillin resistance/β-lactamase (MecI/BlaI) family repressors, based both on the sequence similarity of the N-terminal DNA binding domain and similar 5′-TACAxxTGTA *cop* box palindromic operator sequences (Fig. 1A).^18^ The C-terminal regulatory domain functions as the dimerization domain,^19^ is of unknown structure, and is thought to coordinate both Zn(ii) and Cu(i).

CopY regulates copper efflux and the oxidative stress response in a small number of bacteria from closely related Firmicutes, derived from the *Lactococcus*, *Streptococcus* and *Enterococcus* spp. (Fig. 1B).^20^ CopYs conserve one or two C-terminal CxC (C, Cys; x, any amino acid) motifs that are projected to coordinate Cu(i) or Zn(ii). Zn(ii) is reported to function as an allosteric activator of DNA binding required for full repression of the *cop* operon in the absence of Cu(i) stress. As Cu(i) levels rise in the cell, two Cu(i) have been shown to displace each Zn(ii) to form a luminescent Cu_2_–S_4_ cluster that impairs DNA binding.^28^ This mechanism of allosteric Cu(i)-induced inhibition and Zn(ii)-induced activation of DNA binding is unique among metal efflux regulators, in that the binding of an alternative or non-cognate metal generally has a neutral or similar impact on DNA binding affinities relative to the cognate metal.^21^ Although early studies of CopY have qualitatively characterized the functional outcomes of binding of Zn(ii) *vs.* Cu(i), the extent to which these coordination complexes differ from one another is unknown. This information is needed to elucidate the underlying mechanisms of metal-dependent allosteric activation *vs.* inhibition of DNA binding by CopY and how this impacts *cop* operon expression.

In this work, we present a comprehensive multi-pronged analysis of the different metal-bound states of CopY, particularly in terms of coordination chemistry and metal-induced global structural and dynamical changes. We uncover novel insights into the allosteric activation and inhibition of DNA binding of *S. pneumoniae* CopY by Zn(ii) and Cu(i), respectively, and place these studies in the context of CopY-like repressors that regulate methicillin and β-lactam antibiotic resistance in other human microbial pathogens.^22^–^26^

## Results and discussion

### Coordination chemistry of *Spn* CopY

#### Copper and zinc binding affinities and stoichiometries of *Spn* CopY

CopY from *S. pneumoniae* D39 has high sequence similarity with the previously characterized CopY from *E. hirae*^16^,^27^ and CopR from *L. lactis* (Fig. 1B).^17^,^18^,^20^ This multiple sequence alignment (Fig. 1B) also reveals that C52 and C101 are not conserved in other CopYs, and we therefore use C101A CopY interchangeably with the wild-type protein (*vide infra*). A major finding is that *Spn* CopY retains just two of the four Cys residues near the C-terminus found in all other CopYs. This suggests that the stoichiometry of metal binding in *Spn* CopY may well be distinct from *E. hirae* and other 4-Cys (CxC…CxC)-containing CopYs (Fig. 1B). *E. hirae* CopY binds 2 Cu(i) per protomer or four per dimer,^28^ forming a luminescent binuclear S_4_–Cu_2_ cluster. This is not expected in the case *Spn* CopY, since it lacks the second CxC sequence. In addition, the Zn stoichiometry is 1 per protomer or two per dimer in 4-Cys-containing CopYs, although the identity of the cysteines bound to Zn(ii) is not known.^28^ Zn(ii) is also known to bind *Spn* CopY and is required for complete repression of the *cop* operon in *S. pneumoniae*.^13^ This result suggests that the two conserved Cys are sufficient for binding Zn. However, the Zn stoichiometry for *Spn* CopY has not been measured and little is known about Zn coordination in CopYs. Furthermore, while the DNA binding activities of different CopYs have been investigated,^18^ only relative metal binding affinities have been reported.^28^

**Fig. 2:**
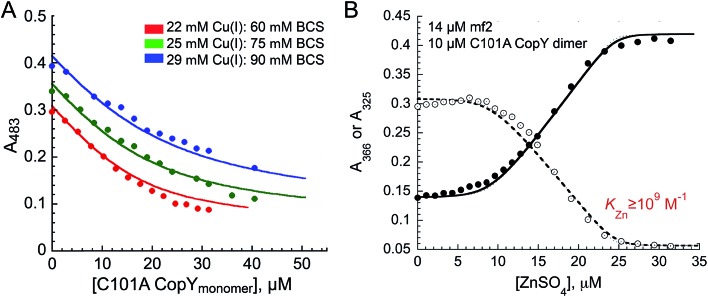
Metal binding affinities of C101A CopY. (A) Representative binding curves obtained from an anaerobic Cu(i) chelator competition to measure the Cu(i) binding affinity of CopY. Apo CopY (monomer units) was titrated into a mixture of Cu(i) and BCS at pH 7.0 in three independent experiments. *Closed* symbols, A483 values for the Cu(i)–(BCS)_2_ complex. The continuous line represents the results of a global analysis of all experiments to a simple 1 : 1 binding model assuming Cu binding sites in each protomer of the homodimer are identical and independent, such that *K*_Cu1_ = *K*_Cu2_ = *K*_Cu_. *Red*, 22 μM CuCl and 60 μM BCS; *green*, 25 μM CuCl and 75 μM BCS; *blue*: 29 μM CuCl and 90 μM BCS, with log *K*_Cu_ = 16.6 (±0.1). (B) Representative Zn(ii) binding curve obtained from a chelator competition assay. ZnSO_4_ was titrated into a solution of apo-C101A CopY (12.0 μM dimer) and mag-fura-2 (mf2) (16 μM) at pH 7.0. Open symbols, A366; closed symbols, A325 for the Zn(ii)-mf2 complex. The continuous line represents the results of a global fit to 1 : 1 (1 Zn : 1 dimer) binding model. Only a lower limit of *K*_Zn_ could be obtained from this experiment, *K*_Zn_ ≥ 10^9^ M^–1^, since the binding is essentially stoichiometric.

The addition of Cu(i) to a solution of apo-CopY with monitoring of Cu–S charge transfer absorption (240 nm (ref. 14 and 29)) reveals saturation at a stoichiometry of 1 per protomer or 2 per dimer (Fig. S1†). The Cu(i) complex is not strongly luminescent at room temperature in aqueous buffers (data not shown), which contrast the findings for 4-Cys containing CopYs and suggests that the cluster is probably solvent exposed and not found within a compact protein environment.^30^ In order to determine the equilibrium constant for Cu binding (*K*_Cu_), we next anaerobically titrated apo-C101A CopY into a mixture of Cu(i) and BCS (*β*_2,Cu_ = 10^19.8^ M^–2^)^31^ and monitored disassembly of the preformed Cu(i) : BCS_2_ complex which absorbs at 483 nm (Fig. 2A). The solid lines represent a simultaneous fit to three different experiments obtained at different Cu(i) and BCS concentrations to two identical Cu(i) sites model on the dimer. These data give log *K*_Cu_ = 16.6 (±0.1). This value is ≈1–2 orders of magnitude weaker than other Cu(i) binding repressors that have been previously characterized, including CsoR and CueR.^8^,^32^,^33^ This result makes the prediction that the concentration of free cytoplasmic Cu(i) in *S. pneumoniae* might be higher than in other pathogens that harbor a Cu(i) sensor from the CsoR or MerR families (see Conclusions).^12^,^21^ Interestingly, the *Spn* CopY Cu(i) binding affinity is comparable to the high affinity site on the N-terminal metal-binding domain of *Spn* CopA^14^ but larger than *K*_Cu_ for Cu chaperone CupA that harbors the functionally essential low affinity Cu(i) binding site (log *K*_Cu_ = 14.8).^14^ This is consistent with a model proposed for other Cu chaperone-Cu-sensing repressor pairs in which the repressor is capable of stripping Cu(i) from the Cu chaperone suggesting that the copper chaperones, transporters and transcriptional repressors have coevolved to maintain this hierarchy in metal affinities.^28^,^34^

We next titrated Zn(ii) into a solution of apo-C101A CopY and the modest affinity chelator mag-fura-2 (mf2; *K*_Zn_ = 5.0 × 10^7^ M^–1^) (see Fig. 2B for a representative experiment), since previous studies on other CopY repressors reveal that active DNA-operator binding form is Zn-CopY.^16^,^28^,^35^ These experiments show that the stoichiometry for Zn(ii) binding is 1 Zn per dimer or 0.5 Zn per protomer, with only a lower limit of *K*_Zn_ ≥ 10^9^ M^–1^ obtained from these experiments. These results reveal that Zn and Cu are likely coordinated by ligands derived from both subunits, contrary to what has been previously proposed for the 4 Cys-containing CopYs^35^ and further suggest that two Cys per protomer are necessary and sufficient to mediate CopY-dependent biological regulation by Cu or Zn.

### X-ray absorption spectroscopy

**Fig. 3:**
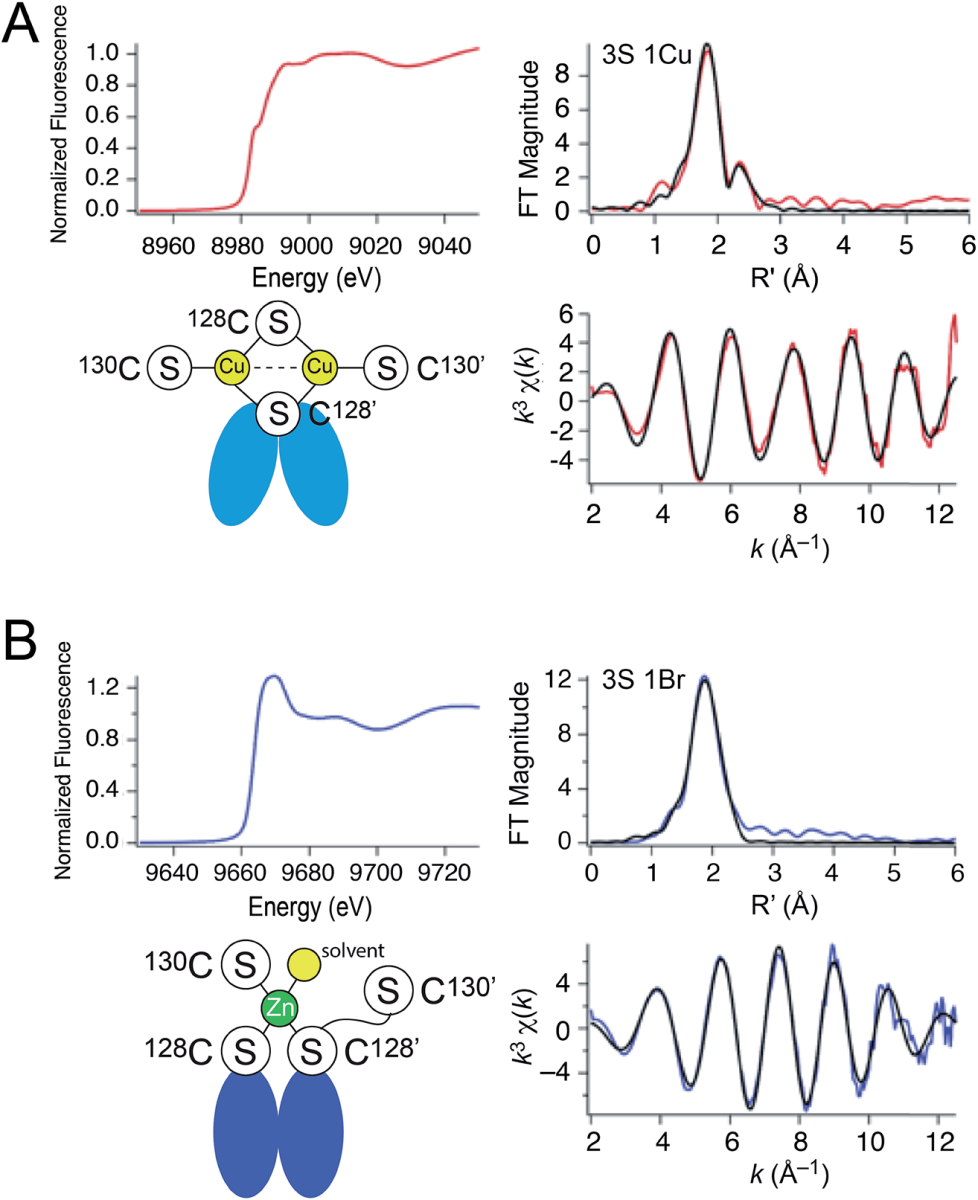
X-ray absorption spectroscopy of (A) Cu_2_ CopY in NaCl-containing buffer (Cu_2_ CopY–Cl) and (B) Zn_1_ CopY acquired in NaBr-containing buffer (Zn_1_ CopY–Br) C101A CopY. In each panel the X-ray absorption near-edge spectrum (XANES) is shown in the *upper left*, the extended X-ray absorption fine structure (EXAFS) and *k*-space spectra shown in *upper right*, and *lower right*, respectively, with cartoon models of each coordination structure consistent with the spectroscopy (*red* continuous lines in the EXAFS and *k*-space spectra, respectively; *black*, best fits to the data) shown in the *lower left*. The Cu(i) XAS spectra are consistent with the same chemical environment around each of the two bound Cu(i) ions containing 3 Cu–S bonds (*d* = 2.27 Å) and one Cu–Cu scatterer (*d* = 2.70 Å), features consistent with the binuclear cluster model shown. The Zn(ii) XAS is consistent with a subunit-bridging cysteine-rich site conforming to a 3S 1H_2_O tetrahedral complex containing 3 Zn–S bonds (*d* = 2.29 Å). See Table 1 for a summary of the best fit parameters and Tables S1–S3† for fits for all possible coordination models.

These metal titrations define the stoichiometry and affinities of Cu(i) and Zn(ii) binding to apo-CopY; however, the metal coordination and ligand identity remain unknown. Thus, we next investigated the coordination structure of the Cu_2_ (1 : 1 protomer; 2 per dimer) and Zn_1_ (0.5 : 1; 1 per dimer) complexes by X-ray absorption spectroscopy (XAS) on the Cu (Fig. 3A) and Zn (Fig. 3B) edges. For the Cu(i) complex, the XANES spectrum is fully compatible with a trigonally coordinated Cu previously reported for 4-Cys containing CopYs.^36^ The 1s → 4p transition has a normalized absorption amplitude of 0.51 at 8982.8 eV for Cu_2_ CopY–Br and 0.52 at 8984.1 for Cu_2_ CopY–Cl. The EXAFS spectrum is well-described by three sulfur scatterers at 2.27 Å and a single Cu–Cu interaction at ≈2.7 Å (Table 1), in general agreement with previous work on *E. hirae* CopY.^28^ These spectra are compatible with a Cu_2_–S_4_ binuclear cluster model shown in which the C-terminal Cys pair (C128, C130 on both protomers) donates the four sulfur ligands to the complex. Despite the fact that single Cu–Cu interaction can also be modeled as a Cu–Br interaction in samples acquired in NaBr-containing buffers (Fig. S2; Table S2†), the Cu–Br bond vector at 2.65 Å (Table S2†) is far too long and we see the same spectrum in the presence of NaCl or NaBr. Thus, we favor the cluster model shown. We note that these spectra cannot be readily distinguished from higher nuclearity clusters of Cu and S, *e.g.*, Cu_4_·S_6_ “adamantane-like” structures common in cysteine sulfur-rich peptides that lack stable secondary structure.^37^,^38^ Since *Spn* CopY harbors only 2 Cys per protomer, the formation of higher nuclearity clusters would induce oligomerization beyond the dimer, and this would be functionally relevant since only the dimer is expected to bind DNA (*vide infra*).

**Table 1:** Fitted parameters obtained from the Cu(i)- and Zn(ii)-bound forms of *S. pneumoniae* CopY^*a*^

Sample	CN	Shell	*r* (Å)	*σ* ^2^ (×10^–3^ Å^–2^)	Δ*E*_o_ (eV)	% *R*
Cu(i), NaBr	3	3 S	2.269(4)	5.0(3)	–6.8(7)	1.44
		1 Cu	2.703(5)	3.9(5)		
Cu(i), NaCl	3	3 S	2.265(5)	5.1(3)	–5(1)	2.82
		1 Cu	2.71(1)	7(1)		
Zn(ii), NaBr	4	3 S	2.29(1)	3.4(5)	–12(2)	0.77
		1 Br	2.48(1)	4(1)		

^*a*^Spectra and fits for the first Cu(i) sample entry and the Zn(ii) entry are shown in Fig. 3 and S2.

The Zn_1_ CopY complex, in contrast, shows a single broad maximum in the post-edge XANES region with normalized intensity of 1.3, consistent with a tetrahedral complex composed of soft ligand donor atoms (Fig. 3B).^39^ The EXAFS analysis is characterized by one or more strong scatterers with no significant outer shell contributions, indicating the absence of ligands such as His. We acquired these spectra in the presence of NaBr rather than NaCl in an effort to probe the Zn(ii) site for the presence of a ligand (Br^–^ or H_2_O) from the solvent, since Cl^–^ is not readily distinguished from S. These data acquired in NaBr-containing buffers are best described by a tetrahedral 3S 1Br complex (Table S3†), in which the conserved C128 and C130 from each of the two subunits are the anticipated thiolate ligands, with one coordination site occupied by Br^–^. This result suggests a solvent-accessible or open coordination site on the Zn(ii). These data provide the first evidence that a change in the coordination number and geometry distinguishes CopY allosteric activation from allosteric inhibition.

### Ratiometric pulsed alkylation mass spectrometry (rPA-MS) of CopY metallostates

The XAS data suggest that one of the two C-terminal Cys (C128 or C130) from one protomer within the dimer is not coordinated in the Zn(ii)-bound state resulting in an open coordination site on the metal (Fig. 3B). In order to confirm this and identify which Cys is not coordinated, we measured the differential reactivity of Cys residues toward an electrophile, *e.g.*, *N*-ethylmaleimide (NEM), as a reporter of the stability of single metal–ligand coordination bonds,^40^,^41^ with metal coordination attenuating the reactivity of the cysteine thiolate anion. We subjected various metallostates of CopY to a pulse of excess *d*_5_-*N*-ethymaleimide (*d*_5_-NEM) for time *t*, following by a chase with a vast excess of protiated NEM (H_5_-NEM) under denaturing conditions, thereby encoding a mass shift of 5.0 Da. Proteins are then digested with a suitable protease, and deuterated and protiated peptides resolved by MALDI-TOF mass spectrometry (Table 2).

**Table 2:** Calculated and observed masses of H_5_-NEM and *d*_5_-NEM-derivatived Lys-C and AspN digested peptides from *Spn* C101A CopY^*a*^

Peptide	Amino acid sequence	Mass (Da)	Cys	Modification	Mass of modified peptides (Da)	
					Calc'd	Obs'd
83-95	DKV**C**SRRIRNLLA	1543.88	C86	H_5_-NEM	1668.93	1668.94
				*d*_5_-NEM	1673.97	1673.99
120-131	SSAVTEVR**C**N**C**M	1299.55	C128, C130	H_5_-NEM/H_5_-NEM	1549.64	1549.71
				*d*_5_-NEM/H_5_-NEM	1554.69	1554.73
				*d*_5_-NEM/*d*_5_-NEM	1559.73	1559.72

^*a*^Carried out as described in Materials and methods with representative data shown in Fig. 4.

**Fig. 4:**
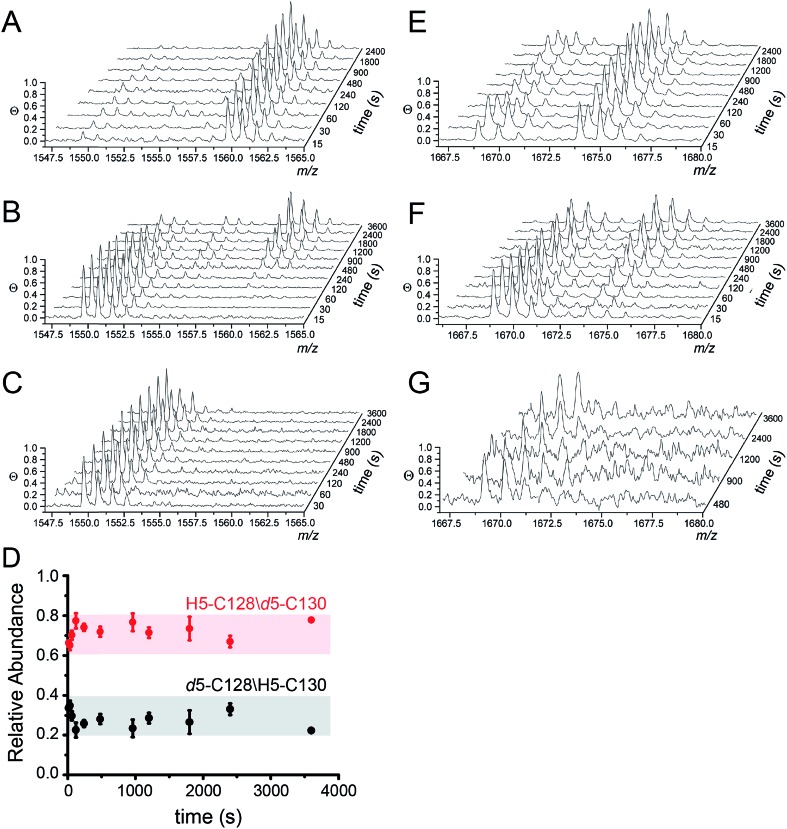
Ratiometric pulsed alkylation-mass spectrometry analysis of *Spn* C101A CopY in the apo-state (A and E), the Zn_1_ (per dimer) state (B and F) and the Cu_2_ (per dimer) (C and G) allosteric states. rPA-MS time course profiles for AspN-derived peptide 120-131 (panels A–C) and LysC-derived peptide 83-95 containing C86 (panels E–G) (see Table 2 for exact masses). (D) Relative quantification of *d*_5_-NEM alkylation events at C128 *vs.* C130 in the Zn_1_ state as a function of *d*_5_-NEM pulse time in the doubly alkylated *d*_5_/H_5_ peptide 120-131 quantified as described in Fig. S3.† Note that C130 is more reactive than C128 at all pulse times.

MALDI-TOF data as a function of pulse time, *t*, for the apo, Zn_1_ and Cu_2_ states reveals C128 and C130 are both highly reactive in the apo-state (Fig. 4A), with nearly full deuteration observed at pulse time of 30 s, and that this reactivity is fully quenched in the Cu_2_ complex (Fig. 4C). In contrast, the Zn complex is kinetically or thermodynamically less stable than the Cu_2_ complex but these Cys are protected relative to the apo-state, revealing detectable singly-deuterated (*d*_5_/H_5_) and double-deuterated (*d*_5_/*d*_5_) NEM-containing peptides at intermediate pulse times, *t* = 240 s (Fig. 4B). This result reveals that Cu binding is fully protective on the cysteine ligands and Zn fails to protect the four cysteine residues involved in Cu(i) coordination.

The results from rPA-MS are consistent with the XAS but do not indicate which Cys residues are more strongly bound to the Zn. This can be determined by subjecting the singly-deuterated (*d*_5_/H_5_) peptide to tandem mass spectrometry (MS/MS) (Fig. S3†). Analysis of these data reveals derivatization of both C128 and C130 in the resulting fragment ions, with the C130-*d*_5_-NEM y- and b-ions consistently accumulating to higher levels that the C128-*d*_5_-NEM ions (Fig. 4D). These data therefore support a model in which C128 from both protomers is strongly coordinated to the Zn(ii), while only one of the two C130 side chains is coordinated in the CopY homodimer (see Fig. 3B).^41^

Additionally, rPA-MS can potentially report on changes in cysteine reactivity in cysteines that are not involved in metal binding but are differentially exposed to solvent. The reactivity of C86 is also somewhat sensitive to metal binding, and is differentially reactive in the Zn- *vs.* Cu-bound states (Fig. 4F and G). Zn slightly attenuates the reactivity of C86 relative to apo-CopY, while Cu(i) appears nearly fully protective. These results suggest that in addition to differences in the first coordination shell, Cu(i) and Zn(ii) may well trigger distinct conformational changes in the CopY dimer.

### DNA-binding properties of the apo-, Zn(ii) and Cu(i)-coordinated CopY

**Fig. 5:**
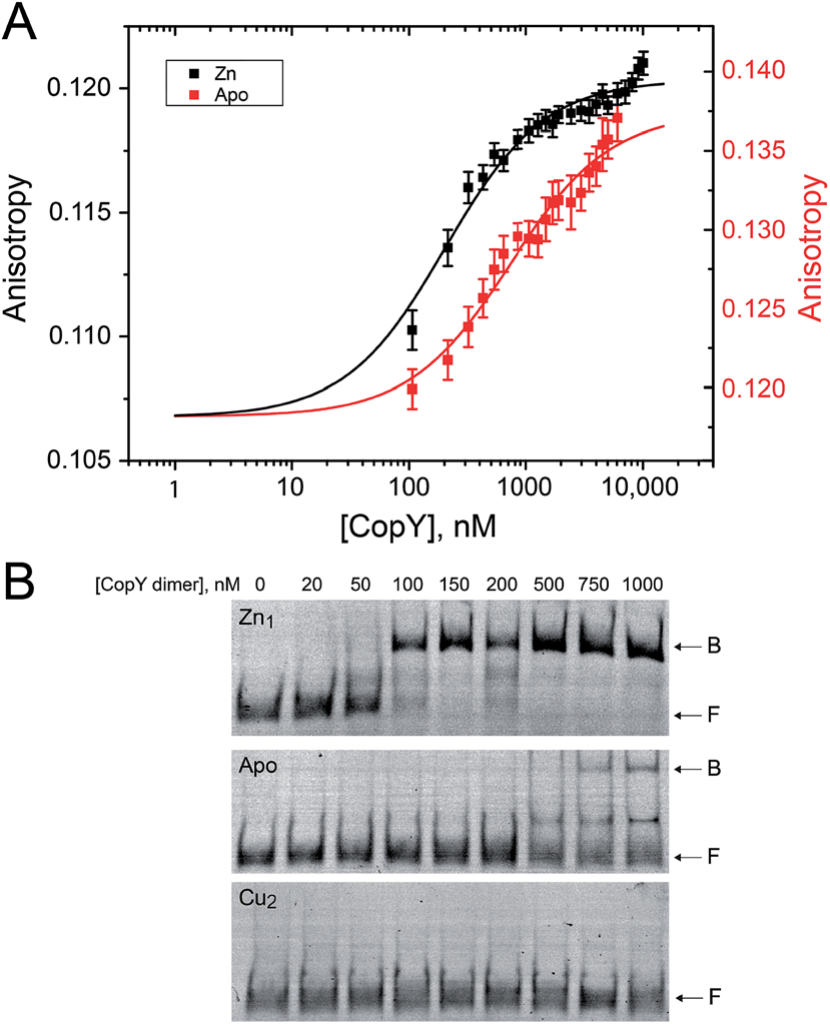
*cop* operator DNA binding activities of various C101A CopY metallostates. (A) Fluorescence anisotropy (FA)-based DNA binding experiment for Zn_1_ (*black*) and apo-states (*red*) of C101 CopY, with the continuous line a fit to 1 : 1 non-dissociable dimer-DNA binding model. *K*_a_ = 1.0 (±0.2) × 10^7^ M^–1^ and Δ*r* = 0.014 (±0.03) for Zn_1_ CopY and *K*_a_ = 2.8 (±0.6) × 10^6^ M^–1^ and Δ*r* = 0.017 (±0.03) for apo-CopY, for Δ*G*_c_ = 0.6 (±0.1) kcal mol^–1^. Conditions: 0.23 M NaCl, 10 mM HEPES, pH 7.0, 2 mM TCEP, 40 μM ZnSO_4_ or 2 mM EDTA, 25 °C. (B) Representative electrophoretic mobility shift analysis (EMSA). *Top*, Zn_1_ C101 CopY dimer; *middle*, apo-state; *bottom*, Cu_2_ C101A CopY. B, bound; F, free DNA. The equilibrium Zn_1_ CopY-dimer DNA binding affinity, *K*_a_, estimated from these data is 2.0 × 10^7^ M^–1^, consistent with the FA-binding data in panel A. Solution conditions: 25 mM HEPES, pH 7.0, 200 mM NaCl (chelexed), 5 mM MgCl_2_, 50 μg mL^–1^ BSA, 25 °C. Apo-CopY samples contained 5 mM EDTA.

With the distinct coordination chemistries of the Zn and Cu-bound states of CopY established by our metal affinity, XAS and rPA-MS experiments, we next aimed to relate these metal binding differences to the *cop* operator DNA binding affinities. Previous work on *E. hirae* CopY suggests that Zn(ii) coordination is essential for DNA binding, while Cu(i) allosterically inhibits DNA binding^28^,^35^ with the latter inducing Cu-dependent transcriptional derepression.^15^ We reconstituted Zn_1_ and Cu_2_ C101A CopYs and measured their DNA binding affinities for a DNA duplex containing a single *cop* box sequence using either a fluorescence anisotropy (FA)-based assay (Fig. 5A)^42^ or an electrophoretic mobility shift assay (EMSA) (Fig. 5B). The FA-based assays reveal that Zn(ii) indeed functions as an allosteric activator of DNA binding, but only increases the apparent affinity ≈4-fold at equilibrium relative to the apo-state, with *K*_a_ ≈ 10^7^ M^–1^ under these conditions (pH 7.0, 0.23 M NaCl, 25 °C). The EMSA experiments largely recapitulate the FA-based assays, except that the apo-CopY complex appears significantly more kinetically labile than the Zn-CopY complex, with only small amounts of DNA complex formation up to 1 μM protein (Fig. 5B). We could observe no complex formation for the Cu(i)-bound state, consistent with allosteric inhibition of *cop* box DNA binding by Cu(i).

### Functional characterization of CopY mutants in cells

**Fig. 6:**
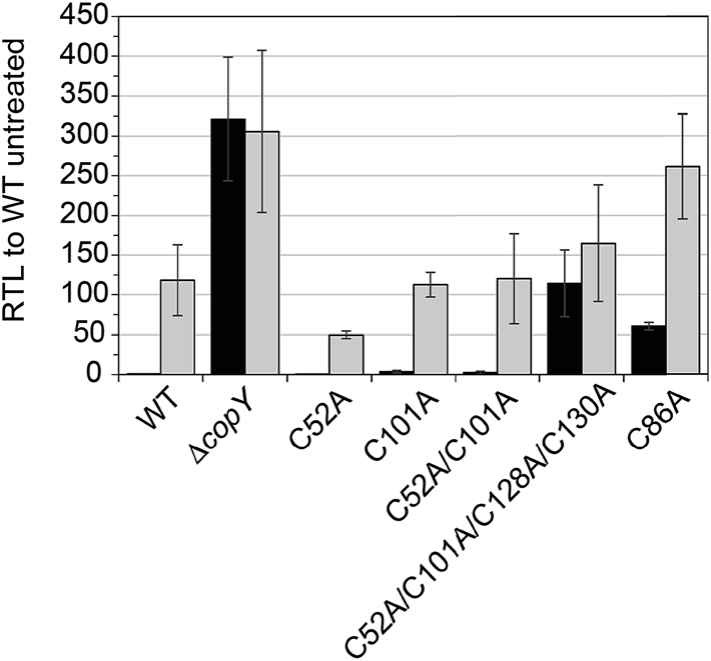
Relative transcript levels (RTL) from qRT-PCR analysis of *copA* transcription. Exponentially growing wild-type (WT), Δ*copY* and *copY* mutant allelic cells were diluted into BHI and allowed to grow to approximately 0.2 OD_620_. Cultures were then spiked with (*grey*) or without (*black*) 200 μM Cu and incubated for 1 h before harvesting cells and RNA extraction. The average and s.d. of three biological replicates are shown.

In order to validate our *S. pneumoniae* CopY coordination models and *cop* operator DNA binding experiments, we constructed *S. pneumoniae* D39 strains harboring mutant CopYs and measured their resistance to cellular copper toxicity (Fig. S4†) and transcriptional regulatory activity (Fig. 6). The addition of 0.2 mM Cu(ii) to the growth medium in mid-log cells results in a ≈100-fold induction of *copA* expression, confirming the Cu-inducibility of the *cop* operon (Fig. 6, WT). In contrast, a Δ*copY* strain is characterized by high level, constitutive *copA* expression that is unchanged by the addition of Cu. Mutant pneumococcal strains harboring C52A, C101A and C52A/C101A *copY* alleles are also strongly Cu-inducible, to an extent similar to the wild-type strain, findings consistent with the non-essentiality of non-conserved cysteines in CopY, C52 and C101. The strain harboring C86A CopY is also Cu-inducible consistent with no direct role in Cu sensing, but gives higher levels of basal *copA* expression (Fig. 6). The differential reactivity or solvent accessibility of C86 in the Zn *vs.* apo allosteric states (see Fig. 4) may be related to this partial loss of repressor function and, thus may be reporting on the importance of the conformation of this linker region for allosteric communication (see Fig. 1). Finally, the quadruple C52A/C101A/C128A/C130A mutant shows no significant Cu regulation, consistent with the requirement that the C-terminal Cys pair (C128, C130) be available to coordinate the allosteric activator Zn, required to obtain full repression of *copA* expression in the absence of Cu stress in cells. All strains grow with WT-like growth kinetics and are unaffected by copper stress, in contrast to the *copA* deletion strain, as expected (Fig. S4†).^14^ Thus, unregulated *cop* operon expression is not deleterious to pneumococcal growth under these growth conditions.

### Structural analysis of CopY metallostates

While the above data clearly show that Zn(ii) and Cu(i) form distinct coordination complexes that oppositely impact *cop* operator DNA binding affinity *in vitro* and in cells, the structural basis for these effects is unknown. In fact, there is no structure available for any full length CopY, with the only high resolution structure of the N-terminal DNA binding domain of *L. lactis* CopR (Fig. 1B).^17^ We therefore investigated the solution structural properties of the apo-, Zn_1_ (1 : 1 per dimer) and Cu_2_ (2 : 1 per dimer) complexes using ^1^H–^15^N NMR spectroscopy, small angle X-ray scattering (SAXS) and ion mobility-mass spectrometry (IM-MS), discussed in turn.

### NMR studies of wild-type and C52A/C101A CopY metallostates

**Fig. 7:**
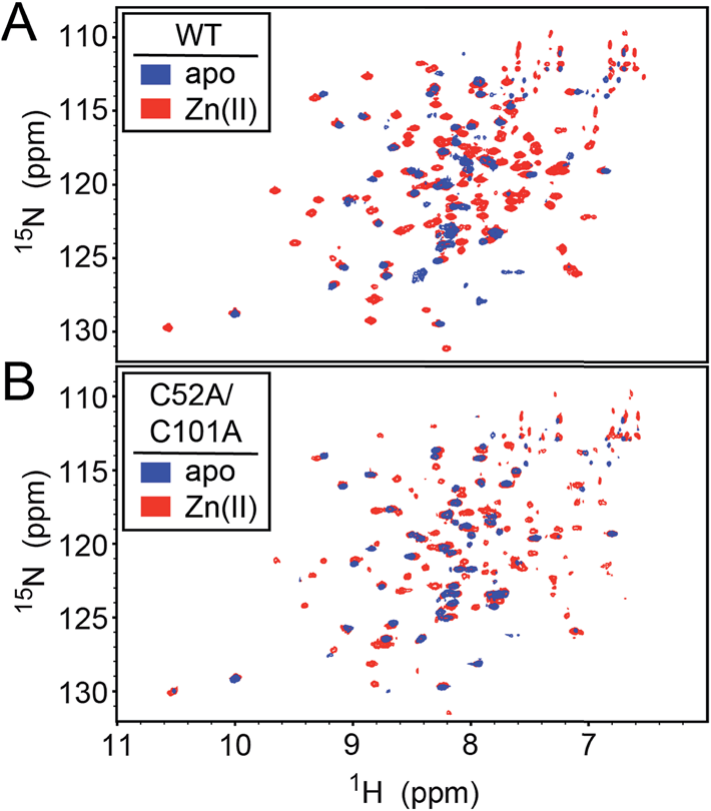
^1^H–^15^N HSQC spectra of intact wild-type (WT) (A) and C52A/C101A (B) *Spn* CopY in the apo-state (*blue* contours) and the Zn_1_ allosteric state (*red* contours). Spectra were acquired at 30 °C in 20 mM HEPES, 0.2 M NaCl, 5 mM TCEP, pH 6.0. Additional NMR spectra of Cu-bound CopY and the C-terminal regulatory domain fragment (68-131) are shown in Fig. S5.†

We first used NMR spectroscopy to characterize the different metallostates of *Spn* CopY, since this method potentially provides both high resolution and site-specific information on both structure and dynamics. ^1^H–^15^N TROSY or ^1^H–^15^N HSQC spectra were acquired for both wild-type and C52A/C101A CopYs (30.9 kDa; Fig. 7). A comparison of the spectra obtained for the apo- and Zn-saturated forms of the repressor reveal that the apoprotein is characterized by considerable conformational dynamics, as very few cross peaks are observed in these spectra. The addition of 0.5 mol protomer·equiv. Zn(ii) sharpens these spectra significantly, and reveals that the structures of the wild-type and C52A/C101A CopYs are substantially identical, consistent with the fact that these two non-conserved Cys are dispensable for CopY function in cells (Fig. 6). However, the relative intensities of the cross peaks vary dramatically even in Zn_1_ CopY and not all anticipated cross peaks are visible, indicative of substantial dynamics in specific regions of the molecule. This severely limits efforts to obtain sequence-specific resonance assignments even with extensively deuterated samples (spectra not shown), which were ultimately unsuccessful. Strikingly, anaerobic addition of 1 mol protomer·equiv. (2 per dimer) Cu(i) to either the apo-CopY or a preformed Zn_1_ CopY dimer results in significant spectral line broadening, far more so than is observed in the apoprotein (Fig. S5†).

In an effort to improve spectral quality, we also acquired ^1^H–^15^N HSQC spectra for what we anticipated would correspond to the C-terminal, metal binding regulatory domain, encompassing residues 68-131 (Fig. S5†). Unfortunately, these spectra, like that of the intact CopY, yielded very little additional information, again due to severe spectral line broadening. The largest number of cross peaks are again obtained with the Zn(ii)-bound regulatory domain, and the majority of the observable cross peaks can be superimposed on those found in the Zn_1_-CopY dimer spectrum. Severe spectral line broadening induced specifically by Cu(i) is fully consistent with some combination of extensive conformational dynamics within a Cu(i)-bridged CopY dimer and oligomerization beyond the dimer to higher order assemblies, also bridged by Cu–S bonds.

### Small angle X-ray scattering (SAXS) analysis

**Fig. 8:**
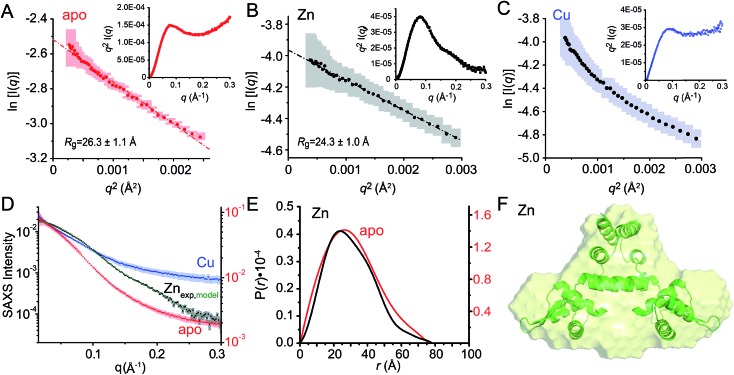
Small angle X-ray scattering (SAXS) analysis of C101A CopY in various allosteric states. Guinier plots and associated linear fits (where possible) of the composite raw scattering curves (panel D) obtained from the apo- (*red*) (A), Zn_1_ (*black*) (B) and Cu_2_ (*blue*) (C) C101 CopYs, with associated Kratky plots shown in the *inset*. (D–F) Quantitative analysis of Zn_1_ C101 CopY. (D) composite scattering curve (log *I vs. q*) for Zn_1_ CopY (*black*), with a curve calculated from the best-fit *ab initio* model (F) (*green*), shown alongside those for apo- (*red*) and Cu_2_ (*blue*) CopYs. (E) Pairwise distribution histogram plot from the data shown in panels A (apo CopY; *red*) and B (Zn_1_ CopY; *black*), with a best-fit DAMMIF *ab initio* model shown for the Zn_1_ CopY (E).^54^ The hydrodynamic parameters obtained for apo- and Zn_1_ CopYs from these data are shown in Table 3.

We next employed small angle X-ray scattering (Fig. 8) in an effort to understand changes in the global shape that occur upon metal coordination. SAXS scattering curves were obtained for the apo-, Zn_1_ and Cu_2_ states, and apo and Zn_1_-states show Guinier plots indicative of monodispersity and a similar radius of gyration (*R*_g_, Fig. 8A and B; Table 3). In contrast, the Guinier plot of the Cu_2_ state shows clear evidence of a heterogeneous sample containing higher order oligomers (Fig. 8C) and as a result, detailed structural analysis of this state was not further carried out. The apo and Zn_1_-states are readily distinguished from one another in the raw scattering profiles (to *q* = 0.5 Å^–1^) as well as in the PDDF plots (*p*(*r*) *vs. r*) (Fig. 8E), with the scattering of the Zn bound state consistent with the prediction based on the previously published crystal structures of BlaI and MecI (Fig. 8D and E). Furthermore, the molecular envelope calculated as an *ab initio* bead model by DAMMIF for the Zn-bound state shows that structure in solution is consistent with a model of CopY based on previously published structures of BlaI and MecI (Fig. 8F), while fully recapitulating the experimental scattering curve (Fig. 8D). Moreover, a qualitative analysis of the Kratky plots suggests that the apo- and Cu_2_ forms of CopY are less structured overall than the Zn bound form (Fig. 8A–C), consistent with the reduced number of cross peaks in the ^1^H–^15^N HSQC spectra of these states relative to the Zn-bound CopY dimer.

**Table 3:** SAXS structural parameters obtained for the apo ad Zn_1_ metallostates of *Spn* CopY^*a*^

	apo CopY	Zn_1_ CopY
*R*_g_ (Å)^*b*^ (Guinier)	26.3 ± 1.1	24.3 ± 1.0
*R*_g_ (Å)^*c*^ (GNOM)	24.3 ± 0.4	22.5 ± 0.6
*D*_max_ (Å)	76	77
MW (kDa)^*d*^	24.7 (30.9)	26.0 (30.9)
MW discrepancy	20.2%	15.9%

^*a*^The SAXS data for the Cu(i) binding metallostate was not further analyzed due to the extreme nonlinearity in the Guinier plots (Fig. 8C).

^*b*^Derived from Guinier fitting.^52^

^*c*^Derived from GNOM analysis.^51^

^*d*^Molecular weight calculated using the MoW2 server,^53^ with theoretical molecular weight calculated from protein sequence (dimer) shown in parentheses.

### Ion mobility-mass spectrometry (IM-MS)

**Fig. 9:**
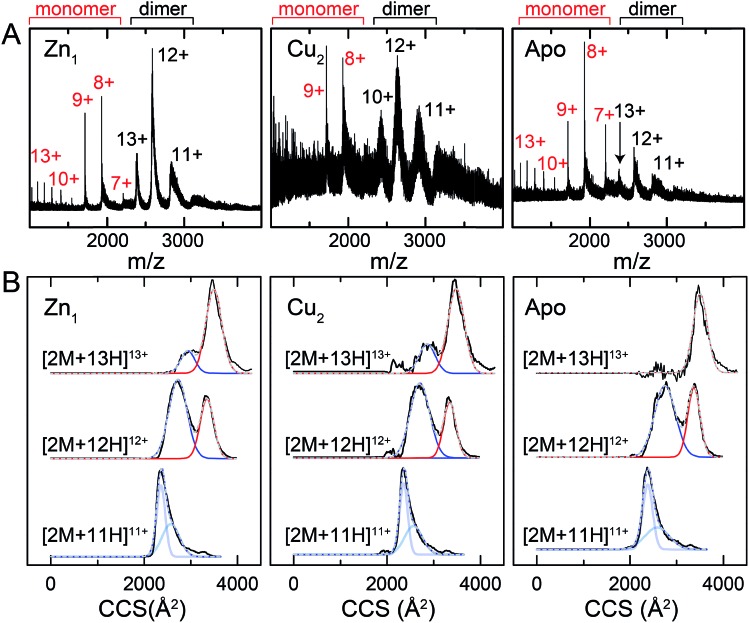
Ion mobility-mass spectrometry (IM-MS) of C101A *Spn* CopY in various allosteric states. Mass (*m*/*z*) spectra (A) and background subtracted mobility distributions for [2M + 11H]^11+^, [2M + 12H]^12+^ and [2M + 13H]^13+^ dimer charge states (B) for the Zn_1_ state (*left*) and Cu_2_ state (*middle*) and apo state (*right*). Monomer (highlighted in *red*) and dimer (*black*) regions are shown in panel A. In panel B, the experimental data are in *black*, and each distribution envelope is fit to the minimum number of Gaussians (*red* or *blue*) required to satisfactorily describe the full envelope (*grey* discontinuous line through the envelope). See Fig. S6† for complete 2D drift plots for these CopY metallostates.

One interpretation of the SAXS and NMR data is that the apo-form of CopY is partially or transiently unfolded, particularly in the C-terminal regulatory domain. To test this, we analyzed the CopY metallostates by ion mobility-mass spectrometry (IM-MS), an experimental technique that can be used to examine partially unfolded states, as recently described for ubiquitin.^43^ We therefore acquired *m*/*z* spectra (Fig. 9A) and ion mobility distributions (Fig. 9B and S6†) for the apo-, Zn_1_- and Cu_2_ complexed forms of CopY that collectively provide insights into monomer stability and mobility profiles of these allosteric states, using methods analogous to those previously applied to the Cu-sensing repressor CsoR.^44^

The mass spectral data (Fig. 9A and S6†) are characterized by three different regions: an area of highly charged monomer from 1000 to 1600 *m*/*z* ([M + 15H]^15+^ to [M + 10H]^10+^), a region of low charged monomer from ≈1600 to 2200 *m*/*z* ([M + 9H]^9+^ to [M + 7H]^7+^) and a region of dimer from 2200 *m*/*z* and greater (principally [2M + 13H]^13+^ to [2M + 11H]^11+^). The relative abundance of the dimer peaks compared to the low-charged monomer peaks reports on the stability of the CopY dimer. For example, in apo-CopY, the low charge states of the monomer, from [M + 9H]^9+^ to [M + 7H]^7+^, dominate the distribution relative to the three readily visible dimer peaks, [2M + 13H]^13+^, [2M + 12H]^12+^ and [2M + 11H]^11+^. On the other hand, the coordination of Zn(ii) to the intersubunit 3S 1H_2_O site results in an increase in the dimer (D) peaks relative to the monomer (M) peaks, with significant suppression of the [M + 8H]^8+^ and [M + 7H]^7+^ charge states. All the monomer (M) peaks are clearly metal-free (as evidenced by the same M masses in each CopY preparation) and this may well derive from electrospray ionization-mediated dissociation of the Zn_1_-CopY complex during transition to the gas phase.^45^ This suggests that Zn binds only to the dimeric CopY, fully consistent with a subunit-bridging coordination model (Fig. 3; *vide infra*). The Cu_2_ CopY complex (Fig. 9A) *m*/*z* spectra are qualitatively similar to that of the Zn-CopY state, except that these spectra are characterized by a very low signal-to-noise (S/N) ratio. Low S/N can either be traced to a low concentration relative to other higher oligomer forms of CopY that predominate in these mixtures (*vide infra*), and/or relatively poor desolvation of the Cu-bound dimer when electrosprayed. Based on the SAXS and NMR analysis, we favor the former interpretation.

Mobility distributions for the [2M + 13H]^13+^, [2M + 12H]^12+^ and [2M + 11H]^11+^ charge states derived from the apo and the metallated CopY dimers are shown in Fig. 9B. Overall, the apo-, Zn_1_ and Cu_2_ states show two major features that correspond to an extended and compact set of conformations, ≈2500 and ≈3200 Å^2^, respectively. The relative abundance of each conformation depends primarily on the charge state as a result of the increase in coulombic repulsion, *i.e.*, the compact conformation is more prevalent in the lower charge states. This columbic repulsion may also explain the small increase in collision cross-section for each conformation with increasing number of charges. Although these gas-phase conformations are not necessarily present in solution, the SAXS and NMR data suggest that CopY exists as a heterogeneous ensemble of different structures, particularly in the apo-state. An extended conformation (≈3400 Å^2^) is indeed more prevalent in the apo-state distributions compared to the Zn_1_-state (Fig. 9B), a finding more evident for the [2M + 13H]^13+^ charge state where the apo-state is exclusively extended. Overall, Zn coordination by the CopY dimer stabilizes a more compact form that likely resembles the SAXS model (see Fig. 8F) and is structurally compatible with DNA binding. In the case of the Cu_2_-metallostate, the dimer is likely not the most abundant oligomeric state in solution, but we could not find any evidence of higher oligomerization states in these ion mobility spectra. These results support the idea that Zn(ii) coordination to the subunit-bridging site restricts access to an extended conformation that is likely partially unfolded and, in this way, enhances DNA binding affinity of the Zn-bound repressor relative to the apo-repressor.

## Conclusions

**Fig. 10:**
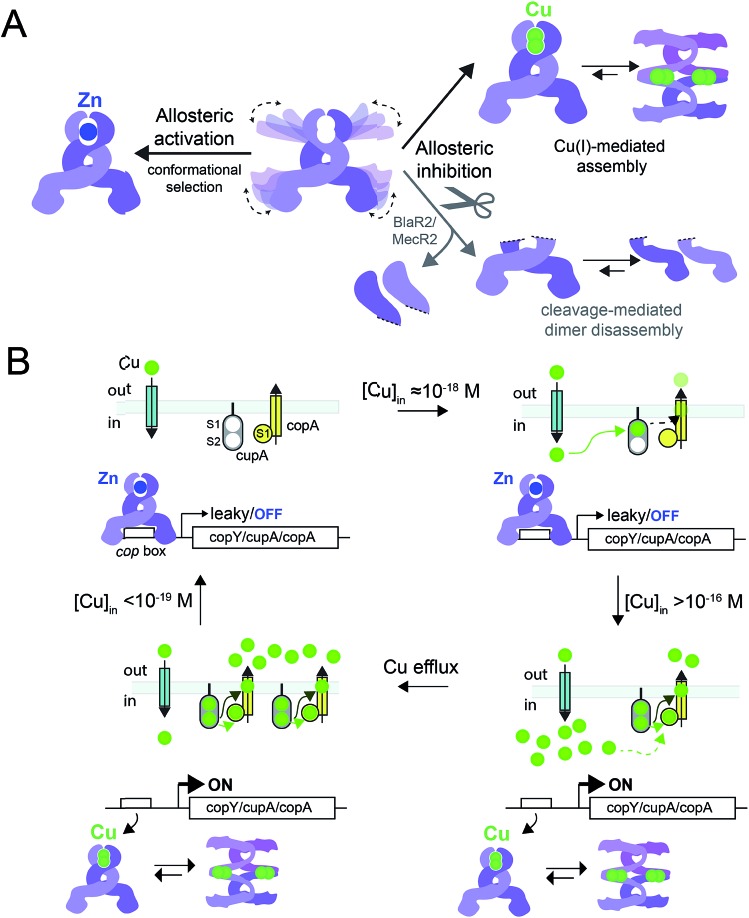
(A) Allosteric model of *Spn* CopY highlighting allosteric activation of DNA operator binding by apo-CopY by Zn, and allosteric inhibition of DNA binding by Cu(i), consistent with the work presented here. We compare metal-dependent CopY regulation with proteolysis-induced regulation of DNA (MecR2; BlaR2) binding by dimeric MecI/BlaI (see text for details). Higher order oligomeric states are represented only as tetramers for clarity. (B) Threshold model for Cu(i) sensing and detoxification in *S. pneumoniae*. The Zn and Cu(i) binding affinities, stoichiometries and coordination structures determined here are placed into the context of previously published work on the membrane-bound Cu chaperone, CupA, and the P-type ATPase efflux pump, CopA.^14^,^55^ The Zn(ii) binding affinity of CopY is such that there is sufficient free Zn in the cell, governed by the relative affinities of the Zn uptake repressor, AdcR,^56^ and the Zn efflux regulator, SczA,^5^ to ensure that any CopY expressed under uninduced conditions will be Zn bound and bound to the *cop* box. As free Cu(i) rises to greater than 10^–16^ M, this is sufficient to trigger Cu binding by CopY (log *K*_Cu_ 16.3) leading to transcriptional derepression; as Cu levels continue to rise, the low affinity S2 Cu site on CupA is filled (log *K*_Cu_ 14.8), which along with CopA is required for cellular Cu resistance. CupA and CopA collaboratively function to lower cellular Cu, and at [Cu] ≤ 10^–19^ M, the regulatory system resets.

In this work, we have determined the Cu and Zn binding affinities, stoichiometries and coordination structures of *Spn* CopY using chelator competition assays, X-ray absorption spectroscopy and rPA-MS. We have also used NMR spectroscopy, SAXS and IM-MS to define the differences in protein structure and dynamics between the metal-free apo state, allosterically activated (relative to DNA binding) Zn(ii) bound CopY, and the allosterically inhibited Cu(i)-bound CopY. Our findings reveal that *Spn* CopY binds up to one dimer mol·equiv. of Zn(ii) or up to two dimer mol·equiv. of Cu(i), respectively. Both metal complexes are subunit-bridging, and involve exclusively coordination by the C-terminal C128–x–C130 pair. There is no spectroscopic evidence for coordination by the C-terminal M131 residue or C86, with the coordination models consistent with mononuclear coordinately unsaturated 3S 1H_2_O Zn(ii) complex and a binuclear Cu_2_–S_4_ Cu(i) or multinuclear Cu_*n*_–S_*m*_ complex (where *n* < *m*). Although XAS data consistent with a binuclear Cu_2_–S_4_ cluster have been previously reported for *E. hirae* CopY,^28^ our SAXS show clear evidence of a heterogeneous sample containing higher order oligomers. This is further supported by the low signal-to-noise in our IM-MS spectra on the Cu_2_ CopY complex, the significant decrease in the number of observable cross peaks in the NMR spectra of this complex, as well as the rPA-MS findings, which reveal that all the solvent-exposed Cys are essentially fully protected by Cu(i) coordination. Although our results cannot clearly distinguish the degree to which Cu-bound oligomers form beyond that of the tetramer, this is the first direct evidence that the metallostructures must be subunit-bridging in any CopY. Further, this work suggests that Cu-mediated oligomerization beyond the dimer is an important aspect of this regulatory process (Fig. 10A).

The average Cu(i) binding affinity of *Spn* CopY, although lower than those measured for other Cu(i)-sensing metalloregulators,^32^,^46^ is consistent with a “threshold” model of cellular Cu resistance defined by a set-point of ≈10^–16^ M free metal in the pneumococcus (Fig. 10B). In this model, the Cu affinity of CopY is tuned such that it is weaker than the high affinity S1 site in metallochaperone CupA, comparable to that of N-terminal metal-binding domain of CopA, but greater than that of functionally critical S2 site in CupA.^14^ In this way, the Cu resistance system is transcriptionally switched “on” at ≈10^–16^ M bioavailable Cu(i), which exceeds the capacity of the existing CupA and CopA molecules produced as a result of “leaky” transcription to buffer Cu(i).^47^ This results in the synthesis of many molecules of CopY, CupA, and CopA, which in turn triggers CupA–CopA-mediated Cu export, and a subsequent drop in cellular Cu to less than 10^–18^ M Cu. A report that appeared during the review of this manuscript which investigates the role of CupA on CopY function in pneumococcal cells under conditions of copper toxicity adds additional detail to this threshold model.^48^

How CopY incorporates allosteric activation of DNA binding by Zn(ii) can be explained by a conformational selection mechanism based on the ion mobility experiments where the population of an extended, partially unfolded, weakly DNA-binding conformation(s) present in the apo state is significantly quenched upon Zn binding. Ligand-dependent activation of DNA binding has been attributed to conformational selection for several transcription regulators that repress or activate gene expression,^49^,^50^ with the catabolite activator protein in *E. coli* the best characterized example. This mechanism has been previously suggested for MecI/BlaI based on the different orientations of the DNA binding motifs in BlaI structures from two different organisms (Fig. S8†).^25^,^26^ However, this idea has not been further evaluated since the regulation of DNA binding of MecI/BlaI is thought to occur mainly *via* selective proteolytic cleavage in the regulatory domain that destabilizes the dimer and dissociates the repressor–operator complex (Fig. 10A).^22^,^23^ In the case of CopY, the weaker DNA binding affinity of the apoprotein is enhanced by Zn coordination at the C-terminal CxC pair, which not only stabilizes the CopY homodimer (Fig. 9) but also selects a compact conformation that it is poised to bind DNA (Fig. 8F). Moreover, the NMR structure of *Lactococcus lactis* CopR^17^ (Fig. 1B) suggests that the relative orientations of the DNA binding helices relative to the rest of the winged helical domain are similar to that found in the intact MecI structure from *S. aureus*^23^ which are not oriented such that the reading heads “fit” into consecutive major grooves (Fig. S7†). This reinforces the idea that conformational plasticity and marginal stability of the regulatory domain must be important aspects of allosteric activation of DNA operator binding of CopY by Zn (Fig. 10A).

Allosteric inhibition by Cu, on the other hand, relies on the formation of a kinetically stable, multi-metallic Cu·S cluster(s) that may well destabilize the dimer by forming higher order oligomers, likely by means of Cu·S·Cu bridges. As discussed above, the mechanism of derepression proposed for MecI posits a proteolysis-dependent change in the stability of the dimer near the residue corresponding to C101 (Fig. 1A); however, in this case, the signaling event (proteolysis) leads to dissociation of dimers to weakly DNA-binding monomers (Fig. 10A).^22^,^23^ On the contrary, Cu leads to the formation of CopY oligomers that are aggregation-prone (Fig. 8C, 10A). It has been shown previously that Cu induces derepression of *E. coli* MarR-regulated genes by a tetramer assembly mechanism;^51^ however, in this case, the oligomers that form are mediated by oxidation of a single Cys in the DNA binding domain to a disulfide, forming crosslinked tetramers and thereby occluding the DNA-binding helices from interacting with the DNA operator. In our CopY model, stable Cu–S coordination bonds may drive assembly beyond the dimer, particularly at excess Cu (Fig. 10B). Our Cu(i)-mediated assembly-inhibition model also readily rationalizes the observation that CopYs contain variable numbers of clustered Cys residues in the C-terminus (Fig. 1B), leading to distinct metal stoichiometries and nuclearities of their multinuclear Cu(i)-thiolate complexes.^28^ All Cu(i) needs to do is drive polynuclear Cu–S cluster formation, sufficient to induce structural and/or dynamical changes in the C-terminal regulatory domain, leading to oligomerization and ultimately DNA dissociation. Zn(ii), in contrast, must be subunit-bridging in a way that stabilizes the dimer and quenches conformational heterogeneity, thereby activating DNA binding. Our finding of a coordinately unsaturated Zn complex likely lowers the kinetic barrier and enhances the rate of metal-exchange between the more weakly bound Zn and more strongly bound Cu(i), thus facilitating transcriptional derepression of the CopY-regulated Cu-resistance genes by increased cellular copper. We propose that nature exploits a marginally kinetically or thermodynamically stable all-α-helical C-terminal regulatory domain on a common N-terminal DNA binding domain as a key feature of CopY and BlaI/MecI-family repressors to effect allostery and biological outputs in distinct ways.

## Methods

See complied ESI.†

## Conflicts of interest

There are no conflicts to declare.

## Supplementary Material

Supplementary information
