# Supplementary material for: Metal-dependent allosteric activation and inhibition on the same molecular scaffold: the copper sensor CopY from Streptococcus pneumoniae
Source: Chem Sci. 2017 Nov 9;9(1):105–18. doi: 10.1039/c7sc04396a (PMC5772342; doi:10.1039/c7sc04396a)
Supplement: Supplementary file 1 [file SC-009-C7SC04396A-s001.pdf]

Electronic Supplementary Material (ESI) for Chemical Science.
This journal is © The Royal Society of Chemistry 2017

ELECTRONIC SUPPORTING INFORMATION (ESI)

Metal-dependent allosteric activation and inhibition on the same molecular scaffold: The copper sensor CopY from *Streptococcus pneumoniae*

Hendrik Glauninger,[a] Yifan Zhang,[a,b] Khadine A. Higgins,[a] Alexander D. Jacobs,[a] Julia E. Martin,[a] Yue Fu,[a,b] H. Jerome Coyne 3rd,[a] Kevin E. Bruce,[c] Michael J. Maroney,[d] David E. Clemmer,[a] Daiana A. Capdevila,[a]* and David P. Giedroc[a,b]*

[a]Department of Chemistry, Indiana University, Bloomington, IN 47405-7102

[b]Department of Molecular and Cellular Biochemistry, Indiana University, Bloomington, IN 47405

[c]Department of Biology, Indiana University, Bloomington, IN 47405

[d]Department of Chemistry, University of Massachusetts, Amherst, MA 01003 USA

This file contains Supplemental Methods, Supplemental Tables S1-S4, and Supplemental Figures S1-S7.

SUPPLEMENTAL METHODS

CopY expression and purification. A pHis plasmid was used to subclone wild-type, C101A and C52A/C101A CopY from *S. pneumoniae* D39 strain (locus tag SPD_0633). This vector encodes a His_6 tag and TEV protease cleavage site at the N terminus to yield, following cleavage, three non-native N-terminal amino acids appended to the sequence GAM for CopY.[1] The same strategy was used to subclone the C-terminal regulatory domain comprising residues 68-131. BL21(DE3) competent cells were transformed with the indicated plasmids. For unlabeled proteins, an overnight culture of *E. coli* was inoculated into Luria Broth (LB) media containing 100 μg/mL ampicillin. For uniformly ^{15}N-labeled or ^{13}C, ^{15}N-labeled proteins, an overnight culture of *E. coli* cells was inoculated into M9 minimal media (pH 7.4) containing 100 μg/mL ampicillin, supplemented with $^{15}NH_4Cl$ (1 g/L) (Cambridge Isotope Laboratories) and when indicated, $^{13}C_6$-*D*-glucose (2.5 g/L) (Cambridge Isotope Laboratories). The cells were typically grown at 37 °C to an $OD_{600} = 0.6$, when isopropyl β-D-1-thiogalactopyranoside (IPTG) was then added to a final concentration of 1 mM and cultures were continued at 16 °C for 20 h. The cells were harvested by low speed centrifugation and kept at –80 °C until use. All the buffers in the purification were degassed and refilled with Argon using a Schlenk line just before use. To break the cells, they were resuspended in buffer R (25 mM Tris, pH 8.0, 200 mM NaCl, 5 mM TCEP) and lysed by a sonic dismembrator (Fisher). The recombinant proteins were purified using Ni-charged HisTrap FF columns (GE Healthcare) using a gradient of imidazole from 25 mM to 325 mM in buffer R. The appropriate fractions were pooled and subjected to TEV protease cleavage at for 16 °C for 36 h and reapplied to the HisTrap FF column, with the flow-through fractions collected and chromatographed on a Superdex 75 16/60 column (GE Healthcare). The purity of the proteins was estimated to be ≥90% as judged by SDS-PAGE and

ESI-MS (Fig. S1A), reduced to a level of ≥90% using Ellman's reagent (DTNB) to detect free thiols[2] and metal-free by atomic absorption spectroscopy or ICP-MS. All CopY preparations contained a trace of what appears to be irreversibly cross-linked dimer on an SDS-PAGE gel, likely the origin of less than fully quantitative recovery of free thiols (Fig. S1A). Protein concentration was determined by A_{280} with an extinction coefficient of 18,240 M^{-1} cm^{-1} on a UV-visible spectrometer (HP/Agilent 8453 spectrophotometer).

Cu(I) binding affinity measurements. Bathocuproine disulfonate (BCS) was used for Cu(I) binding affinity determination of C101A CopY essentially as previously described.[1] All proteins for binding experiments were buffer exchanged from protein stock to the degassed buffer B (25 mM HEPES, pH 7.0, 200 mM NaCl) in an anaerobic glove box. Cu(I) stock was prepared by taking the supernatant from the mixture of CuCl powder with fully degassed buffer B (25 mM HEPES, pH 7.0, 200 mM NaCl), with the concentration of Cu(I) stock determined by atomic absorption spectroscopy (Perkin-Elmer) with a concentration generally around 10 mM. The final titration solution contains 20–30 μM Cu(I) and 60–90 μM BCS in Buffer B. Each 120 μL aliquot of titration solution was mixed with increasing CopY titrant. Optical spectra of BCS were recorded from 200 nm to 900 nm. Corrected spectra were obtained by subtracting baseline spectrum from each CopY addition spectrum, and then corrected for dilution. A_{483} was used to determine the concentration of Cu_2-BCS complex with an extinction coefficient of 13,500 M^{-1} cm^{-1}.[3] All the data were fitted to the appropriate competition model using Dynafit[4] as previously described.

Zn(II) binding affinity measurements. These experiments were carried out as described previously using absorption spectroscopy (A_{325} for apo-mf2; A_{366} for Zn(II)-mf2) of mag-fura-2 (mf2).[5,6] Briefly, anaerobic titrations of a $ZnSO_4$ stock into a solution of a known concentration

of apo-C101A CopY and mf2 were carried out and the resultant data fit to a 1:1 (Zn:CopY dimer) chelator competition model using Dynafit[4], assuming a non-dissociable CopY dimer. Conditions were generally 25 mM HEPES, 200 mM NaCl, pH 7.0 (chelexed before use), 2 mM TCEP, and a ≈1:1 mixture of CopY monomer and chelator (≈15 μM each).

X-ray absorption spectroscopy. C101A CopY was used for all of these experiments. Apo-CopY samples were incubated with 0.5 protomer mol equivalents (1 per dimer) Zn(II) (Zn_1 CopY), or 1 protomer mol equivalents (2 per dimer) Cu(I) prepared in NaBr (Cu_2 CopY–Br) or NaCl (Cu_2 CopY–Cl) were concentrated to 800 μM, 1.9 mM and 2.4 mM protomer, respectively, in 25 mM HEPES, pH 7.0, 200 mM NaCl or NaBr. The samples were prepared under anaerobic conditions. 60 μL of each sample was syringed into polycarbonate XAS holders that were wrapped in kapton tape and frozen in liquid nitrogen. XAS data were collected for individual samples as indicated under dedicated ring conditions on beamline X3b at the National Synchrotron Light Source (NSLS), Brookhaven National Laboratories or on beamline 7-3 at Stanford Synchrotron Radiation Laboratory (SSRL). Samples at the NSLS (Zn_1 CopY–Br and Cu_2 CopY–Cl) were loaded into an aluminum sample holder, which was cooled to ~50 K by using a He displex cryostat. Data were collected under ring conditions of 2.8 GeV and 120-300 mA using a sagitally focusing Si(111) double-crystal monochromator. Harmonic rejection was accomplished with a Ni-coated focusing mirror. X-ray fluorescence was collected using a 30-element Ge detector (Canberra). Scattering was minimized by placing a Z-1 filter between the sample chamber and the detector. Data at the SSRL (Cu_2 CopY–Br) were collected at 10 K using a liquid helium cryostat (Oxford Instruments) with ring conditions of 3 GeV and 80-100 mA. Beamline optics consisted of a Si(220) double-crystal monochromator and two rhodium-coated mirrors, a flat mirror before the monochromator for harmonic rejection and vertical

columnation, a second toroidal mirror after the monochromator for focusing. X-ray fluorescence was collected using a 30-element Ge detector (Canberra). Scattering was minimized by using Soller slits and placing a Z-1 filter between the sample chamber and the detector. XANES were collected from ± 200 eV relative to the metal edge. The X-ray energy for each metal K_α-edge was internally calibrated to the first inflection point of the corresponding metal foil for Cu, 8980.3 eV, and Zn, 9660.7 eV. EXAFS was collected to 15 *k* above the edge energy (E_o).

XAS Data Reduction and Analysis. The XAS data shown (*vide infra*) are the average of 5 and 7 scans for Zn_1 CopY and Cu_2 CopY, respectively. XAS data were analyzed using SixPack.[7] The SixPack fitting software builds on the ifeffit engine.[8,9] Each data set was background-corrected and normalized. The EXAFS equation is defined as (eq 1):

$$\chi(k) = \sum_i \frac{N_i f_i(k) e^{-2k^2\sigma_i^2}}{k r_i^2} \sin[2kr_i + \delta_i(k)] \quad (1)$$

where *f(k)* is the scattering amplitude, *δ(k)* is the phase-shift, *N* is the number of neighboring atoms, *r* is the distance to the neighboring atoms, and σ^2 is the disorder to the nearest neighbor. For EXAFS analysis, each data set was converted to *k*-space using the relationship (eq 2):

$$k = \left[2m_e(E - E_o)/\hbar^2\right]^{1/2} \quad (2)$$

where m_e is the mass of the electron, $\hbar$ is Plank's constant divided by 2π, and E_o is the threshold energy of the absorption edge. The threshold energy chosen for copper is 8990 eV and zinc is 9670 eV.[10] The best fits for the data sets were obtained using a Fourier-transform over the range k = 2-12.5 Å^{-1}, where the upper limit was determined by the signal:noise ratio.

Scattering parameters were generated using FEFF 8.[9] The first coordination sphere was determined by setting the number of scattering atoms in each shell to integer values and systematically varying the combination of S, Cl, Br-donors (Tables S1-S3). To compare different models of the same data set, ifeffit utilizes three goodness of fit parameters: χ^2, reduced χ^2, and

the R-factor. χ^2 is given by eq 3, where N_{idp} is the number of independent data points, $N_{\varepsilon 2}$ is the number of uncertainties to minimize, $\mathrm{Re}(f_i)$ is the real part of the EXAFS function, and $\mathrm{Im}(f_i)$ is the imaginary part of the EXAFS fitting function (eq 3).

$$\chi^2 = \frac{N_{idp}}{N_{\varepsilon^2}} \sum_{i=1}^{N} \left\{ [\mathrm{Re}(f_i)]^2 + [\mathrm{Im}(f_i)]^2 \right\} \quad (3)$$

Reduced $\chi^2 = \chi^2 / (N_{ind} - N_{\mathrm{var}ys})$ where N_{varys} is the number of refining parameters and represents the degrees of freedom in the fit. Additionally, Ifeffit calculates the R-factor for the fit, which is given by eq 4, and is scaled to the magnitude of the data making it proportional to χ^2 (eq 4).

$$R = \frac{\sum_{i=1}^{N} \left\{ [\mathrm{Re}(f_i)]^2 + [\mathrm{Im}(f_i)]^2 \right\}}{\sum_{i=1}^{N} \left\{ [\mathrm{Re}(\tilde{x}data_i)]^2 + [\mathrm{Im}(\tilde{x}data_i)]^2 \right\}} \quad (4)$$

In comparing different models, the R-factor and reduced χ^2 parameter were used to determine which model was the best fit of the data. The R-factor will always generally improve with an increasing number of adjustable parameters, while reduced χ^2 will go through a minimum and then increase, indicating that the model is over-fitting the data.[11]

Ratiometric Pulsed-Alkylation Mass Spectrometry (rPA-MS). *Alkylation step.* Sample preparation was adapted from an earlier report[12] and optimized for C101A CopY. All the experiments ware performed anaerobically in a glovebox. The purified C101A CopY was first buffer exchanged into buffer C (25 mM HEPES pH 7.0, 200 mM NaCl, 5 mM EDTA) to remove the reducing reagent and any bound metal ions in the protein stock. Apo state C101A CopY were prepared in Buffer D (25 mM HEPES pH 7.0, 200 mM NaCl) by a second round of buffer exchange. The Cu(I) and Zn(II) forms of C101A CopY were prepared by incubating a

slight excess of Zn (1.2 dimer equivalents) or Cu(I) (2.4 dimer equivalents) with the apo C101A CopY for 1 h, followed by a buffer exchange into Buffer D to remove the unbound metal ions. The pulsed alkylation reactions were carried out in buffer D in a total reaction volume of 600 μL. The pulsed alkylation reagent d_5-*N*-ethylmaleimide (*d*5-NEM, Isotech, Inc.) was dissolved in acetonitrile at 25 mM. Apo C101A CopY (monomer concentration of 50 μM) was allowed to react with a 3-fold thiol excess of *d*5-NEM. At different pulsed time intervals (*t*), a 50 μL aliquot was removed and added to 50 μL of chase solution containing a 900-fold thiol excess of protiated *N*-ethylmaleimide (H5-NEM), 100 mM Tris-HCl, pH 8.0, and 8 M urea. After a 1 h chase, the quenched reaction was removed from the anaerobic chamber and precipitated by adding a final concentration of 12.5% trichloroacetic acid (TCA) on ice for 1.5 h. The precipitated protein pellets were collected by centrifugation at 4 ºC for 30 min, with the supernatant discarded and the pellet washed by iced acetone three times. After the final wash, the pellet was dried by speed-vacuum centrifugation at 45 ºC for 10 min. The same experiments were performed with the Zn_1 C101A CopY and Cu_2 C101 CopY but with a modified chase solution condition containing either 10 mM EDTA (for Zn CopY) or 10 mM bicinchoninic acid (BCA) (for Cu CopY), to dissociate any cysteine-metal bonds during the chase step.

Protease digestion and MALDI-TOF Mass Spectrometry. Both Lys-C and Asp-N proteases were individually applied to produce the dominant peptides contain either [C86] or [C128+C130]. For the Lys-C digestion, the vacuum dried protein pellets were re-suspended in 20 μL digestion buffer (20 mM ammonium bicarbonate, 10% acetonitrile, 2 M Urea, pH 8.0) with 100 ng Lys-C added. For the Asp-N digestions, protein pellets were also re-suspended in 20 μL digestion buffer (50 mM Tris-HCl, pH 8.0, 2.5 mM Zinc Sulfate) with 100 ng Asp-N added. Both protease digestions were performed under 37 ºC for 20 h. Protease digestions were

quenched by adding 2 μL 10% trifluoroacetic acid (TFA) and the digestion products first purified by C-18 Zip-tip before spotting on MALDI-plate with α-cyano-4-hydroxycinamic acid (CCA) matrix by a 5:1 matrix:sample ratio for mass spectrometry analysis. The MALDI-TOF mass spectra data of all the samples were collected by using a Bruker Autoflex III MALDI-TOP mass spectrometer with 200 Hz frequency-tripled Nd:YAG laser (335 nM).

Tandem LC-ESI-MS/MS of C101A CopY. In the MALDI-TOF spectra of the C128/C130-containing Lys-C peptide, 120-131 (SSAVTEVRC̲NC̲M) obtained for the Zn_1 C101A CopY, a peak containing a single *d*5-NEM modification peptides was observed. To identify the site(s) of *d*5-NEM modification, the *d*5/H5 modified peptide was subjected to LC-ESI-MS/MS and the fragmentation pattern of the alkylated peptide at each pulse time *t* was determined by integration of the tandem MS/MS spectra. These data were quantified by summing the fragment intensity to obtain the mol fraction of the d_5-modification on either C128 or C130. The mol fraction of the *d*5/H5 peptide is a combination of C128 and C130 reaction products defined by molar ratio in the MS/MS.

Electrophoretic mobility shift analysis (EMSA) of CopY-*cop* operator DNA binding equilibria. Various C101A CopY metallostates (apo-, Zn_1 and Cu_2 dimers) formed as described in the X-ray absorption spectroscopy section of the Materials and Methods were added to 10 nM 32-bp fluorescein (**F̲**) labeled *cop* operator DNA fragment, 5'**F̲**-GTC TAT AAT TG**A C**A**A** A**TG T**AG ATT TTA AGA GC-3' and its complement (the ACA-xx-TGT *cop* box is highlighted) at the indicated concentrations in 25 mM HEPES, pH 7.0, 200 mM NaCl (chelexed), 5 mM $MgCl_2$, 50 μg/mL BSA, 25 ºC. Apo samples also contained 2 mM EDTA in the samples, as well as in the gel running buffer and the gel itself. Samples were incubated for 30 min and loaded onto a pre-run native PAGE (10%) gel run at 80 V at 4 ºC in a pH 7.5 running

buffer (89 mM boric acid, 89 mM Tris base). Gels were subjected to fluorescence imaging using a Typhoon Imaging system.

Fluorescence anisotropy. Standard fluorescence anisotropy-based DNA binding experiments were also carried out using the same **F**-DNA under similar solution conditions (10 mM HEPES, pH 7.0, 0.23 M NaCl, 2 mM TCEP, 40 μM $ZnSO_4$ or 0.5 mM EDTA, 25 ºC) exactly as described in previous work.[13] All EMSA and anisotropy-based data were fit to a simple 1:1, non-dissociable dimer binding model to estimate K_a using DynaFit.[4]

Bacterial strain construction, growth conditions for *S. pneumoniae*. The strains in the study were derived from *Streptococcus pneumoniae* D39 (IU1781) and are listed in Table S4. All *S. pneumoniae* D39 mutants were constructed by gene deletion replacement and counter antibiotic selection using the *rpsL*+ cassette, Janus[14] as previously described using standard techniques.[15] Brain-heart infusion (BHI) medium was of standard composition and prepared with double distilled water. For growth experiments, bacteria were inoculated into BHI broth from frozen culture stocks, then serially diluted and propagated overnight. The next day, exponentially growing cultures were diluted to approximately 0.005 OD_{620} into pre-warmed BHI containing increasing concentrations of $CuSO_4$. All aerobic cell growth experiments were monitored over time at 37 °C in an atmosphere of 5% CO_2.

RNA isolation and quantitative real-time PCR (qRT-PCR). Total RNA was isolated from cells by hot phenol extraction, DNase digested and converted to cDNA as previously described.[6] PCR amplification was carried out as previously described using the *copA* forward and reverse primers. *gyrA* served as the housekeeping gene.[16] PCR outcomes were normalized to the *gyrA* gene and relative transcript levels were calculated by comparison of the ratio of stressed to non-stressed cells.

NMR spectroscopy. Typical NMR sample solution conditions were 300-600 µM ^{15}N- or $^{15}N/^{13}C$-labeled wild-type, C101A or C52A/C101A CopY in 20 mM HEPES, 0.2 M NaCl, 5 mM TCEP, pH 6.0 as indicated. All NMR samples were prepared in an anaerobic glove box. Apo-CopY was prepared with 5 mM TCEP. Agilent 800 and 600 MHz spectrometers equipped with cryogenic probes in the METACyt Biomolecular NMR Laboratory were used to acquire data for all CopY samples. NMR data were processed using NMRPipe and were analyzed using Sparky[17]. All spectra were acquired at 25 °C or 30 ºC as indicated. Chemical shift is referenced relative to 2,2-dimethyl-2-silapentene-5-sulfonic acid (DSS).

Small angle x-ray scattering (SAXS). The apo, Zn_1 and Cu_2 binding states of C101A CopY were prepared using the same protocol as described in the rPA-MS experiments. All CopY protein samples were prepared in 25 mM HEPES, 200 mM NaCl, 2mM TCEP at three different protein concentrations (5 mg/mL, 2.5 mg/mL and 1.25 mg/mL). The scattering data were obtained at sector 12ID-B at the Advanced Photo Source (APS) at Argonne National Laboratory. The buffer scattering was subtracted from the sample scattering, with modified sample scattering curves merged to generate the scattering curve for further analysis. The scattering data at low q values are used to estimate the radius of gyration (R_g) by the Guinier approximation with the range of qR_g <1.3. A proper range of data points with q between zero and $3/R_g$ were selected to generate the real space pair distance distribution function (PDDF) using GNOM. D_{max} were carefully calibrated in order to make the PDDF curve drop smoothly to zero. A model of Zn_1 CopY was constructed using DAMMIF[18] that superimposes well with a SWISS MODEL-generated model of CopY from *S. aureus* BlaI.[19]

Electrospray Ion Mobility-Mass Spectrometry (IM-MS). Fully reduced and metal-reconstituted C101A CopY samples for IM-MS were buffer-exchanged into 100 mM ammonium

acetate using Amicon® Ultra–0.5 3k microfilter devices (Millipore, Billerica, MA). This process was repeated six times to remove any salts and buffers incompatible with electrospray ionization (ESI). Final sample concentrations were 10 µM dimer. IM-MS data were recorded on a Synapt G2S HDMS instrument (Waters Corporation, Manchester, United Kingdom). Instrument conditions were optimized for the transmission of large non-covalent structures and metal bound proteins.[20-22] Briefly, samples were introduced into the source of the instrument at a flow rate of 30 µL/h using a kD Scientific syringe pump (KD Scientific Inc. Holliston, MA). Samples were ionized through ESI in positive ion mode, with a source voltage of 2.00 kV. Sampling cone and source offset were set to 30.0 V and 50.0 V respectively. Source and desolvation temperatures were set to 25 °C. Trap gas was set to 2.00 mL/min. IMS cell gas was set to 90 mL/min. Trap and transfer cell wave velocity and wave height were 150 m/s and 8.0 V respectively. IMS wave velocity and wave height were 250 m/s and 20.0 V respectively. Collisional cross section calibration was performed with ubiquitin, cytochrome c and myoglobin.[20] The coefficient of determination was 0.997 with calculated error of species of known collision cross section to be <0.5%. Mass spectra and mobility distributions were analyzed using Mass Lynx v4.1 (Waters Cooperation, Manchester, United Kingdom) and OriginPro 2016 (OriginLab Cooperation, New Hampton, MA). The IM spectra shown in Fig. 9 were background subtracted integrating the signal before each peak and fitted to Gaussian peaks.

SUPPLEMENTAL TABLES

Table S1. Selected EXAFS fits for Cu_2 CopY dimer in buffer containing NaCl. Data fit from k = 2-12.5 $Å^{-1}$ and r = 1–4 Å.

N	r (Å)	σ^2 (x10^{-3} $Å^2$)	ΔE_o (eV)	R factor	χ^2	Reduced χ^2
2S	2.261(9)	2.6(5)	-6(2)	0.0774	1286.42	76.58
3S	2.260(9)	5.2(5)	-6(2)	0.0779	1294.79	77.08
4S	2.26(1)	7.5(8)	-7(2)	0.1266	2104.35	125.27
5S	2.26(2)	1.0(1)	-8(2)	0.1940	3222.89	191.86
1S	2.21(4)	2(4)				
1S	2.29(3)	0(3)	-7(3)	0.0692	1150.96	77.77
2S	2.265(9)	2.6(7)				
1S	2.40(7)	15(15)	-3(2)	0.0634	1054.58	71.26
2S	2.270(8)	2.5(6)				
2S	2.44(4)	17(7)	-1(2)	0.0589	979.43	66.18
3S	2.285(8)	4.5(5)				
1S	2.52(2)	4(2)	-2(1)	0.0440	731.06	49.40
2S	2.266(6)	2.5(4)				
1Cu	2.71(2)	9(2)	-4(1)	0.0409	680.21	45.96
**3S**	**2.265(5)**	**5.1(3)**				
**1Cu**	**2.71(1)**	**7(1)**	**-5(1)**	**0.0282**	**468.73**	**31.67**
4S	2.265(8)	7.3(6)				
1Cu	2.71(2)	6(2)	-6(1)	0.0669	1112.66	75.19
2S	2.258(6)	2.6(4)				
1Cl	2.87(2)	5(2)	-6(1)	0.0389	646.31	43.67
2S	2.263(8)	2.7(6)				
1Cl	2.37(9)	18(18)	-4(3)	0.0638	1061.3	71.72
3S	2.257(7)	5.1(4)				
1Cl	2.87(2)	5(2)	-7(1)	0.0435	722.66	48.83
3S	2.284(8)	4.5(5)				
1Cl	2.51(2)	5(2)	-2(2)	0.0497	825.72	55.80

4S	2.26(1)	7.5(7)				
1Cl	2.87(3)	5(3)	-8(2)	0.0972	1614.98	109.13
4S	2.291(8)	7.0(6)				
1Cl	2.53(1)	2(1)	-2(1)	0.0525	872.59	58.96

Table S2. Selected EXAFS fits for Cu_2 CopY dimer in buffer containing NaBr. Data fit from k = 2-12.5 Å^{-1} and r = 1–4 Å.

N	r (Å)	σ^2 (x10^{-3} Å^2)	ΔE_o (eV)	R factor	χ^2	Reduced χ^2
2S	2.26(1)	2.4(7)	-7(3)	0.1530	9740.66	579.87
3S	2.26(1)	5.0(8)	-8(2)	0.1550	9866.13	587.34
4S	2.26(2)	7(1)	-9(3)	0.1973	12561.61	747.81
5S	2.26(2)	10(1)	-9(3)	0.2547	16211.81	965.11
1S	2.21(8)	3(8)				
1S	2.28(5)	0(4)	-9(5)	0.1423	9062.09	612.39
2S	2.29(1)	1.7(7)				
1S	2.50(3)	4(3)	-1(3)	0.1187	7556.37	510.64
2S	2.29(1)	1.5(6)				
2S	2.49(2)	9(3)	1(2)	0.1010	6428.81	434.44
3S	2.25(2)	5(1)				
1S	2.07(9)	21(23)	-10(4)	0.1516	9649.92	652.11
2S	2.270(6)	2.5(4)				
1Cu	2.71(1)	4.7(9)	-6(1)	0.0396	2524.62	170.60
**3S**	**2.269(4)**	**5.0(3)**				
**1Cu**	**2.703(5)**	**3.9(5)**	**-6.8(7)**	**0.0144**	**921.28**	**62.25**
4S	2.269(6)	7.3(5)				
1Cu	2.702(7)	3.4(7)	-7(1)	0.0364	2321.66	156.89
2S	2.274(8)	2.6(5)				
1Br	2.65(1)	6(1)	-5(2)	0.0551	3508.83	237.11
3S	2.275(4)	5.2(3)				
1Br	2.651(5)	4.7(5)	-6.3(8)	0.0146	932.34	63.00

4S	2.276(5)	7.4(4)				
1Br	2.651(5)	4.0(5)	-6.8(8)	0.0217	1381.56	93.36

Table S3. Selected EXAFS fits for Zn_1 CopY dimer in buffer with NaBr. Data fit from k = 2-12.5 Å^{-1} and r = 1–2.5 Å.

N	r (Å)	σ^2 (x10^{-3} Å^2)	ΔE_o (eV)	R factor	χ^2	Reduced χ^2
2S	2.32(2)	1(1)	-6(5)	0.1379	1369.05	201.42
3S	2.33(1)	3.0(7)	-7(3)	0.0581	576.74	84.85
4S	2.326(9)	4.6(6)	-7(2)	0.0350	347.72	51.15
5S	2.33(1)	6.1(7)	-8(2)	0.0456	453.27	66.68
2S	2.29(4)	2(4)				
1S	2.38(6)	0(6)	-7(4)	0.0459	455.78	95.01
2S	2.26(2)	0.6(14)				
2S	2.37(2)	0(2)	-8(2)	0.0213	212.14	44.22
3S	2.36(2)	2(2)				
1S	2.25(4)	0(3)	-6(3)	0.0295	293.40	61.16
2S	2.27(1)	0.6(7)				
1Br	2.47(2)	1.9(9)	-13(3)	0.0186	185.59	38.69
2S	2.27(2)	2(1)				
2Br	2.46(2)	6(1)	-14(4)	0.0222	220.49	45.96
**3S**	**2.29(1)**	**3.4(5)**				
**1Br**	**2.48(1)**	**4(1)**	**-12(2)**	**0.0077**	**77.24**	**16.10**
4S	2.31(2)	6.0(8)				
1Br	2.46(2)	6(2)	-10(2)	0.0121	120.27	25.07

Table S4. Bacterial strains and plasmids used in this study

Strain	Genotype	Reference
S. pneumoniae D39		
IU1781	D39 *rpsL1* (Str^R)	[23]
IU3525	D39 *rpsL1* Δ*copY::*[Kan^R *rpsL*$^+$]	This work
IU3566	D39 *rpsL1* Δ*copY* (Str^R)	This work
IU6418	D39 *rpsL1* Δ*copY*-(C52A) (Str^R)	This work
IU6420	D39 *rpsL1* Δ*copY*-(C101A) (Str^R)	This work
IU6422	D39 *rpsL1* Δ*copY*-(C52A/C101A) (Str^R)	
IU7576	D39 *rpsL1* Δ*copY*-(C52A/C101A/C128A/C130A) (Str^R)	This work
IU13337	D39 *rpsL1* Δ*copY*-(C86A) (Str^R)	This work
IU5976	D39 *rpsL1* Δ*copA (SPD_0635)* (Str^R)	[1]
E. coli		
DH5α	F^- *endA1 glnV44 thi-1 recA1 relA1 gyrA96 deoR nupG purB20* ϕ80d*lacZ*ΔM15 Δ(*lacZYA-argF*)U169 hsdR17(r_K^- m_K^+), λ^-	Laboratory stock
Rosetta (DE3)	F^- *ompT hsdS*$_B$(r_B^- m_B^-) *gal dcm* (DE3)	Laboratory stock

Plasmid	Relevant characteristics	Reference
pHis.Parallel1	Protein expression vector, Amp^R	[24]
pHis_CopY	pHis.Parallel1 with the *copY gene (SPD_0633*)	This work
pHis_CopY_C101A	pHis.Parallel1 with the *C101A copY gene (SPD_0633*)	This work
pHis_CopY_C52A_C101A	pHis.Parallel1 with the *C52A/C101A copY gene (SPD_0633*)	This work
pHis_CopY_Cterm	pHis.Parallel1 with the *residues 68-131 of copY gene (SPD_0633*)	This work

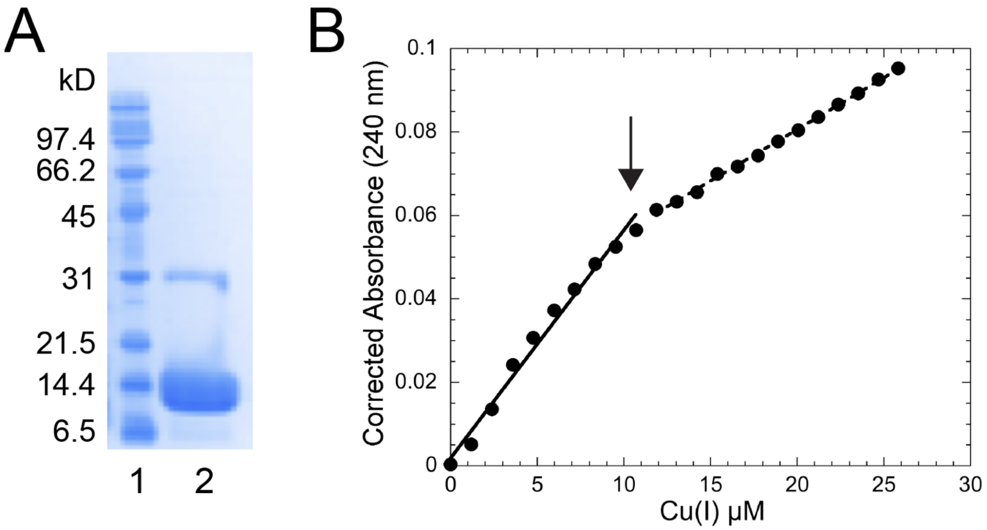

Figure S1. Initial characterization of *Spn* C101A CopY. (A) SDS-PAGE of apo-C101 CopY as purified. Lane 1, Molecular weight standards; lane 2, 10 µg of purified C101A CopY. (B) Anaerobic titration of 10 µM apo-wild-type CopY protomer with copper(I) as monitored by LMCT band plotted at A_{240}. Conditions: 10 mM HEPES, 200 mM NaCl, pH 7.0, at ambient temperature. This experiment defines the Cu(I) binding stoichiometry of 1:1 Cu:CopY protomer or 2 per dimer (Cu_2•$CopY_2$).

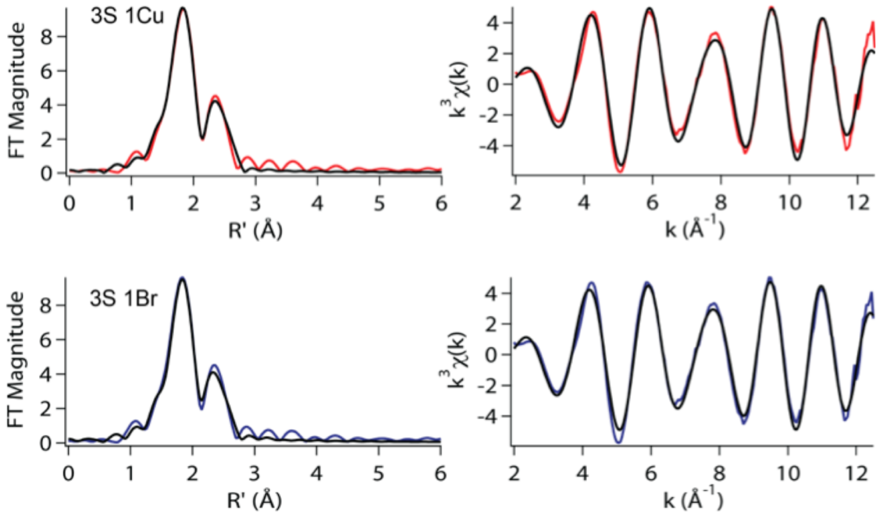

Figure S2. XAS analysis of the Cu_2-C101A CopY dimer acquired in 0.2 M NaBr fitted to two different coordination models. EXAFS, *left*; *k*-space scattering spectra, *right*. *Top*, 3S, 1Cu model (*black* continuous line, data; *red* continuous line, fit); *bottom*, 3S, 1Br model (*black* continuous line, data; *blue* continuous line, fit). See Tables 1 and S2 for a summary of the fitted parameters. The fact that the Cl^--containing sample gives a nearly indistinguishable spectrum provides strong support for the bi- or multinuclear cluster model (see Fig. 3A, main text) containing a Cu-Cu scattering component rather than a Cu-Br component, which are not readily experimentally distinguished from one another. However, a Cu-Br bond vector of 2.65 Å (Table S2) is unreasonably long (compare to 2.48 Å for Zn-Br bond; Table 1), thus providing additional support for assignment of the 2.65 Å component to a Cu-Cu scattering component.

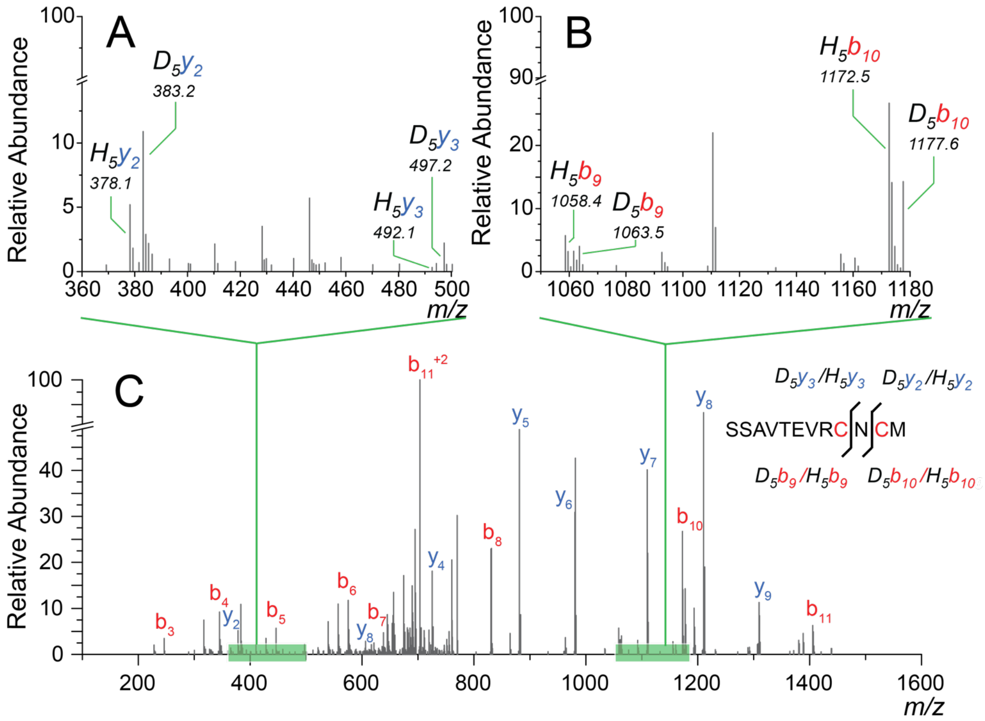

Figure S3. Spectral enlargements (A, *m/z* 360-500; B, *m/z* 1050-1180) of the (C) tandem ESI-MS/MS spectrum of peptide 120-131 SSAVYEVRC̲NC̲M (*m/z*=1554) derived from Zn_1 C101 CopY at pulse time, *t*=2400 s. The relative abundances of deuterated (*d*5) and protiated (H5) (*b*9 + *y*3) and (*b*10 + *y*2) ions were used to quantify the molar ratio of each alkylation intermediate, *d*5-C128/H5-C130 or H5-C128/*d*5-C130, respectively, with the results shown in Fig. 4D (main text). C130 is more likely to be alkylated during all *d*5-NEM pulse times.

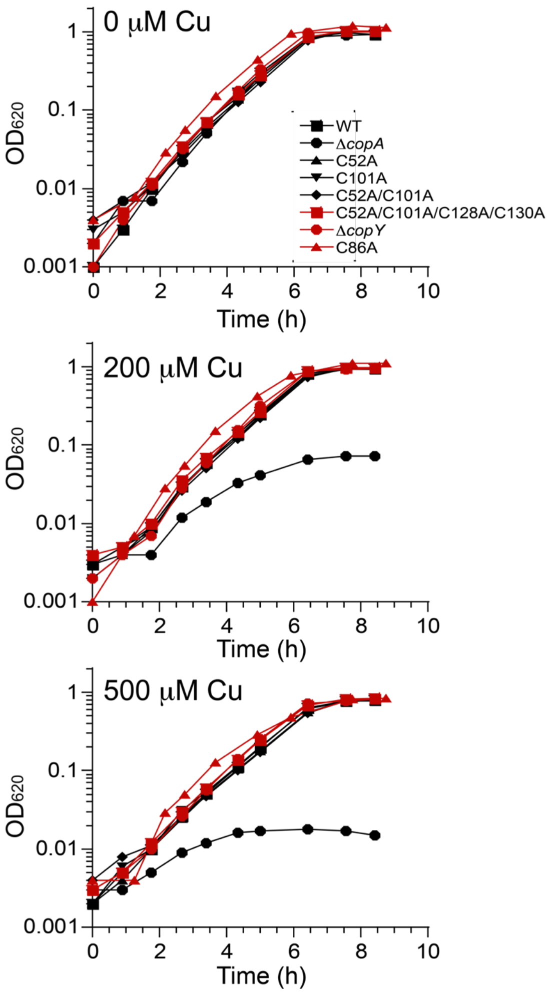

Figure S4. Exponentially growing cells were diluted into BHI with 0 (A), 200 (B), or 500 (C) μM Cu at *t*=0 and growth was followed over time. WT, wild-type D39 strain; Δ*copA*, deletion of the copper-transported ATPase,[1] Δ*copY*, deletion of the copper-sensing repressor. All other strains harbor mutant alleles of *copY* as indicated in standard one-letter code. Only the Δ*copA* strain has a growth phenotype, consistent with unregulated *cupA/copA* expression as having no impact on cell viability at low or high Cu. See Table S4 for details on strain construction.

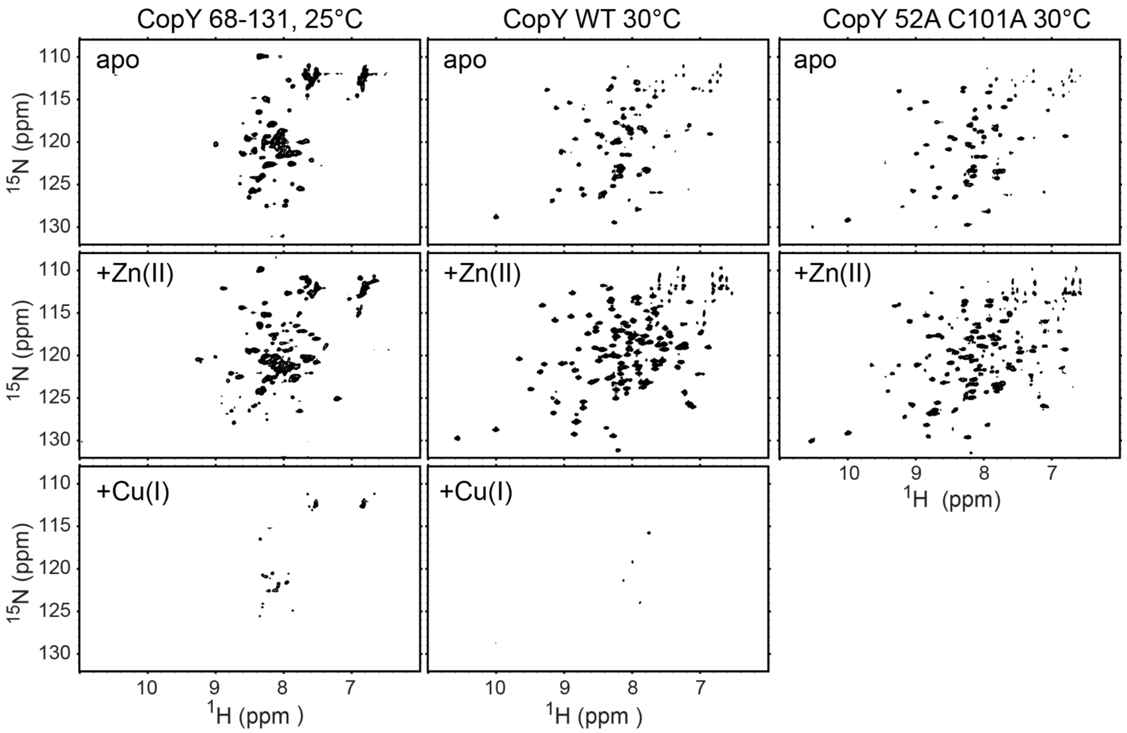

Figure S5. ^{1}H-^{15}N HSQC spectra of different metallated states of the C-terminal metal binding domain of CopY, CopY$^{68-131}$ (*left*), intact wild-type (WT) CopY (*middle*) and the fully functional (see Fig. 8) double mutant of CopY, C52A/C101A CopY (*right*). Apo-proteins are shown at the *top*, following the addition of 0.5 protomer-equivalents of the Zn(II) (*middle*), or following the addition of 1.0 protomer-equivalents of Cu(I) (bottom). Solution conditions: 25 mM HEPES, 0.2 M NaCl, 5 mM TCEP, pH 6.0 at the indicated temperature.

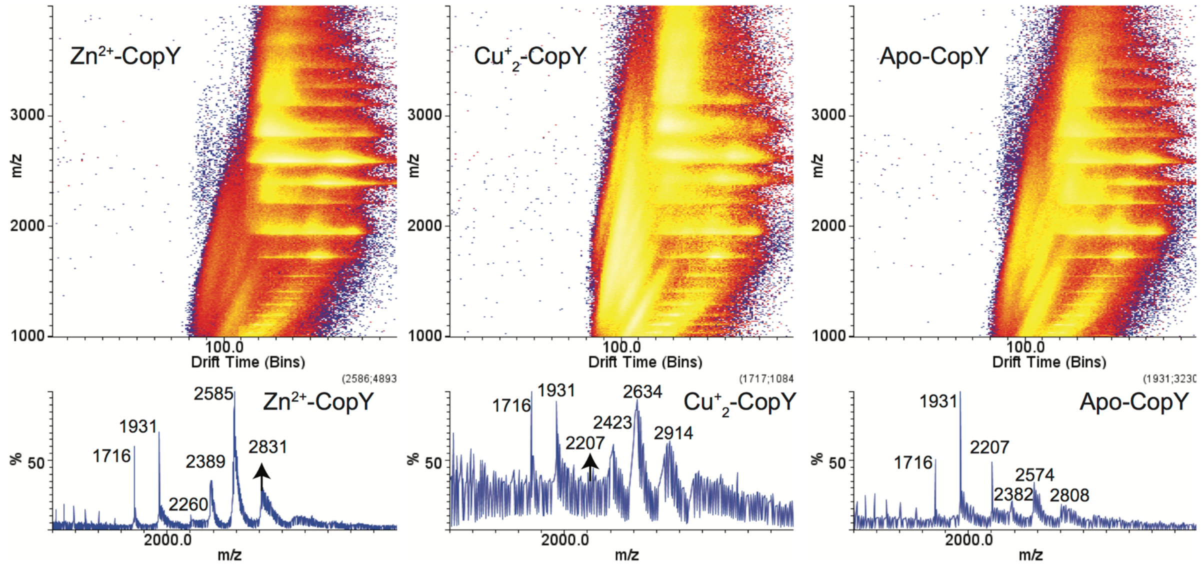

Figure S6. IM-MS analysis of the various metallostates of C101A CopY. *Top,* 2D (drift time, millisecond vs. *m/z*) plots obtained for the Zn_1 (Zn^{2+}-CopY), Cu_2-bound (Cu^{+}_2-CopY) and apo forms of dimeric CopY. *Bottom*, corresponding charge state spectra of the three CopY metalloforms (see Fig. 9A, main text for a more extensively annotated version of these data).

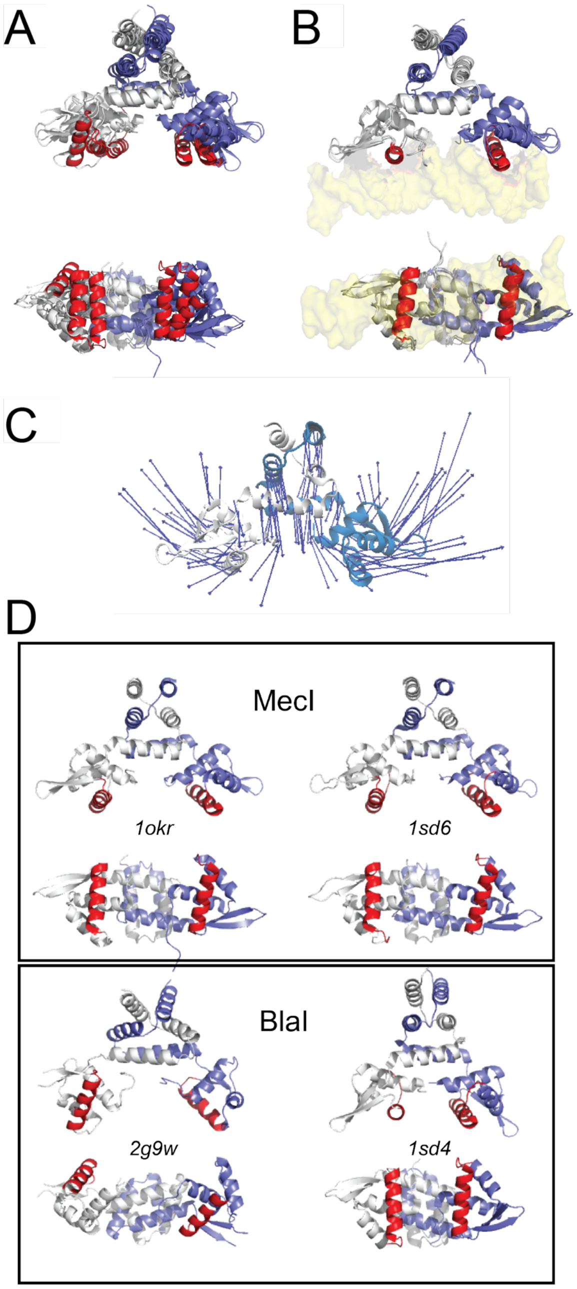

Figure S7. Structural comparison of CopY-like repressors that regulate pathogen β-lactam resistance. Ribbon representations of *Mycobacterium tuberculosis* and *Staphylococcus aureus* BlaI and *Staphylococcus aureus* MecI free in solution (A) and bound to their respective DNA operators (B). In all models, one protomer is shaded *blue*, the other shaded *white*, with the DNA-binding recognition helixes colored *red*., and the bound DNA shown in transparent spacefill *yellow*. Side views (*top*) and bottom views from the perspective of the DNA-binding helices (*bottom*) are shown. (A) Global superposition of the free *S. aureus* BlaI (pdb code 1sd4; 2.0 Å resolution),[19] *S. aureus* MecI (1okr; 2.4 Å resolution),[25] *S. aureus* MecI (1sd6; 2.65 Å resolution)[19] and *M. tuberculosis* BlaI (2g9w; 1.8 Å resolution).[26] (B) Global superposition of the DNA operator-bound *S. aureus* MecI (2d45; 3.8 Å resolution),[27] *S. aureus* MecI (1sax; 2.8 Å

resolution)[28] and *S. aureus* BlaI (1xsd, 2.7 Å resolution).[19] (C) Normal mode[29] analysis of the free *S. aureus* MecI homodimer (1sd6), which reveals that pushing "down" on the top of the C-terminal regulatory domain, moves the DNA-binding helices outward, and out of register with DNA. (D) Another view of the free *S. aureus* MecI (*top*), and *S. aureus* and *M. tuberculosis* BlaI (2g9w) repressors in the same orientation and shading as in panel (A), with pdb codes indicated. A comparison of these structures suggests significant conformational heterogeneity in the free repressors that is essentially lost upon DNA binding (see text for details).

SUPPLEMENTAL REFERENCES:

1. Y. Fu, H.-C. T. Tsui, K. E. Bruce, L.-T. Sham, K. A. Higgins, J. P. Lisher, K. M. Kazmierczak, M. J. Maroney, C. E. Dann, M. E. Winkler and D. P. Giedroc, *Nat Chem Biol* 2013, **9**, 177-183.
2. M. Zimmermann, O. Clarke, J. M. Gulbis, D. W. Keizer, R. S. Jarvis, C. S. Cobbett, M. G. Hinds, Z. Xiao and A. G. Wedd, *Biochemistry*, 2009, DOI: 10.1021/bi901573b.
3. Z. Xiao, J. Brose, S. Schimo, S. M. Ackland, S. La Fontaine and A. G. Wedd, *The J Biol Chem*, 2011, **286**, 11047-11055.
4. P. Kuzmic, *Anal Biochem*, 1996, **237**, 260-273.
5. J. E. Martin, J. P. Lisher, M. E. Winkler and D. P. Giedroc, *Mol Microbiol*, 2017, **104**, 334-348.
6. J. E. Martin, K. A. Edmonds, K. E. Bruce, G. C. Campanello, B. A. Eijkelkamp, E. B. Brazel, C. A. McDevitt, M. E. Winkler and D. P. Giedroc, *Mol Microbiol*, 2017, **104**, 636-651.
7. S. M. Webb, *Physica Scripta*, 2005, **T115**, 1011-1014.
8. S. I. Zabinsky, J. J. Rehr, A. Ankudinov, R. C. Albers and M. J. Eller, *Phys Rev B Condensed Matter*, 1995, **52**, 2995-3009.
9. A. L. Ankudinov, B. Ravel, J. J. Rehr and S. D. Conradson, *Phys Rev B*, 1998, **58**, 7565-7576.
10. S. Leitch, M. J. Bradley, J. L. Rowe, P. T. Chivers and M. J. Maroney, *J Am Chem Soc*, 2007, **129**, 5085-5095.
11. R. W. Herbst, A. Guce, P. A. Bryngelson, K. A. Higgins, K. C. Ryan, D. E. Cabelli, S. C. Garman and M. J. Maroney, *Biochemistry*, 2009, **48**, 3354-3369.
12. J. L. Apuy, X. Chen, D. H. Russell, T. O. Baldwin and D. P. Giedroc, *Biochemistry*, 2001, **40**, 15164-15175.
13. D. A. Capdevila, J. J. Braymer, K. A. Edmonds, H. Wu and D. P. Giedroc, *Proc Natl Acad Sci U S A*, 2017, **114**, 4424-4429.
14. C. K. Sung, H. Li, J. P. Claverys and D. A. Morrison, *Appl Environ Microbiol*, 2001, **67**, 5190-5196.
15. H. C. Tsui, D. Mukherjee, V. A. Ray, L. T. Sham, A. L. Feig and M. E. Winkler, *J Bacteriol*, 2010, **192**, 264-279.
16. K. M. Kazmierczak, K. J. Wayne, A. Rechtsteiner and M. E. Winkler, *Mol Microbiol*, 2009, **72**, 590-611.
17. T. D. Goddard and D. G. Kneller, UCSF
18. D. Franke and D. I. Svergun, *J Appl Crystallogr*, 2009, **42**, 342-346.

19. M. K. Safo, Q. Zhao, T. P. Ko, F. N. Musayev, H. Robinson, N. Scarsdale, A. H. Wang and G. L. Archer, *J Bacteriol*, 2005, **187**, 1833-1844.
20. B. T. Ruotolo, J. L. Benesch, A. M. Sandercock, S.-J. Hyung and C. V. Robinson, *Nat Prot*, 2008, **3**, 1139-1152.
21. R. Salbo, M. F. Bush, H. Naver, I. Campuzano, C. V. Robinson, I. Pettersson, T. J. Jørgensen and K. F. Haselmann, *Rapid Commun Mass Spectrom*, 2012, **26**, 1181-1193.
22. A. D. Jacobs, F. M. Chang, L. Morrison, J. M. Dilger, V. H. Wysocki, D. E. Clemmer and D. P. Giedroc, *Angew Chem Int Ed Engl*, 2015, **54**, 12795-12799.
23. S. Ramos-Montanez, H. C. Tsui, K. J. Wayne, J. L. Morris, L. E. Peters, F. Zhang, K. M. Kazmierczak, L. T. Sham and M. E. Winkler, *Mol Microbiol*, 2008, **67**, 729-746.
24. P. Sheffield, S. Garrard and Z. Derewenda, *Protein Expr Purif*, 1999, **15**, 34-39.
25. R. Garcia-Castellanos, A. Marrero, G. Mallorqui-Fernandez, J. Potempa, M. Coll and F. X. Gomis-Ruth, *J Biol Chem*, 2003, **278**, 39897-39905.
26. C. Sala, A. Haouz, F. A. Saul, I. Miras, I. Rosenkrands, P. M. Alzari and S. T. Cole, *Mol Microbiol*, 2009, **71**, 1102-1116.
27. M. K. Safo, T. P. Ko, F. N. Musayev, Q. Zhao, A. H. Wang and G. L. Archer, *Acta Crystallogr*, 2006, **62**, 320-324.
28. R. Garcia-Castellanos, G. Mallorqui-Fernandez, A. Marrero, J. Potempa, M. Coll and F. X. Gomis-Ruth, *J Biol Chem*, 2004, **279**, 17888-17896.
29. S. P. Tiwari, E. Fuglebakk, S. M. Hollup, L. Skjaerven, T. Cragnolini, S. H. Grindhaug, K. M. Tekle and N. Reuter, *BMC Bioinform*, 2014, **15**, 427.
